# A Cardiovascular Care Pathway Supported by Remote Patient Management in Dutch Primary Care: Cost-Effectiveness, Budget Impact, and Workload Analysis

**DOI:** 10.2196/79512

**Published:** 2026-09-28

**Authors:** Jan Heijdra Suasnabar, Margot Rakers, Nicoline van Hattem, Eric Hiddink, Marcel Haas, Xuerui Zhang, Douwe Atsma, Tobias N Bonten, M Elske van den Akker-van Marle, Hendrikus J A van Os

**Affiliations:** 1 Section of Medical Decision Making, Biomedical Data Science, Leiden University Medical Center, Albinusdreef 2, Leiden, 2333 ZB, The Netherlands, 31 71 526 45 74; 2 Department of Public Health and Primary Care, Leiden University Medical Center, Leiden, The Netherlands; 3 National eHealth Living Lab (NeLL), Leiden University Medical Center, Leiden, The Netherlands; 4 Stichting Health Base, Houten, The Netherlands; 5 Health Campus, Leiden University Medical Center, The Hague, The Netherlands; 6 Department of Clinical Epidemiology, Leiden University Medical Center, Leiden, The Netherlands; 7 Department of Industrial Design Engineering, Delft University of Technology, Delft, The Netherlands; 8 Department of Cardiology, Leiden University Medical Center, Leiden, The Netherlands; 9 Department of Psychiatry, University Medical Center Utrecht, Utrecht, The Netherlands

**Keywords:** patient management, preventive cardiology, primary care, cost-effectiveness, budget impact

## Abstract

**Background:**

Remote patient management (RPM) that supports patient self-monitoring of vital parameters and lifestyle factors may improve cardiovascular risk management (CVRM) in primary care. However, large-scale implementation remains limited, partly due to insufficient evidence on long-term value for money, budget impact, and implications for health care professionals' workload.

**Objective:**

This study aimed to estimate the long-term cost-effectiveness, 5-year health care budget impact, and expected changes in general practitioner (GP) and practice nurse (PN) workload associated with the CVRM-Box intervention in the Netherlands.

**Methods:**

We conducted a model-based economic evaluation comparing CVRM-Box with care as usual in Dutch primary care integrated CVRM programs. CVRM-Box is a multicomponent RPM intervention comprising a digital blood pressure (BP) monitor, digital weight scale, step counter/activity tracker, and a mobile app, with measurements transferred to the GP practice for periodic review. A time-inhomogeneous cohort Markov model simulated lifetime transitions among health states, including at-risk, post–myocardial infarction (MI), poststroke, recurrent events, cardiovascular death, and noncardiovascular death. Cardiovascular risks were modeled using prediction equations (SCORE2, SCORE2-OP, and SMART2) populated with subgroup-specific risk factor profiles. Intervention effects were modeled as changes in systolic BP derived from a matched cohort study of CVRM-Box. Other key parameters (costs and utilities) were similarly obtained from the matched cohort study, or routine primary care data, and published sources. Outcomes included incremental cost-effectiveness ratios (ICERs, indicating cost per quality-adjusted life year [QALY] gained), cost-effectiveness probabilities, 5-year health care budget impact, and an exploratory workload analysis estimating annual changes in visit time and remote consultation frequency for GPs and PNs. Probabilistic and scenario uncertainty analyses were performed.

**Results:**

In the overall population, CVRM-Box increased costs and QALYs versus care as usual, yielding an ICER of €17,340/QALY gained (EUR €1=US $1.11 as of 29 December 2023) and a 60% probability of cost-effectiveness at a willingness-to-pay threshold of €20,000/QALY. Cost-effectiveness was more favorable in higher-risk subgroups (uncontrolled BP and/or prior MI/stroke), with ≥70% probability of cost-effectiveness at €20,000/QALY, whereas in the lower-risk subgroup with controlled BP and no prior MI/stroke, the intervention is unlikely to be cost-effective (ICER: €42,384/QALY). The 5-year health care budget impact was €2.9 million and €662.2 million for regional and national rollout, respectively. In a typical Dutch primary care practice, the CVRM-Box reduced PN workload by 25.9 h and 44.7 remote consultations annually, while the change in GP workload was negligible (0.5 h; 3.3 remote consultations).

**Conclusions:**

The CVRM-Box multicomponent RPM intervention is likely to be cost-effective among high-risk subgroups but not in lower-risk groups. The intervention further reduces PN workload but not GP workload. Results primarily apply to the Dutch context and may not generalize to health care systems with different payment/incentive arrangements. Additionally, long-term outcomes were modeled using prediction models rather than observed cardiovascular events. Nonetheless, our findings provide an argument for implementation in primary care for higher-risk subgroups.

## Introduction

Cardiovascular disease (CVD) is a leading cause of death globally, contributing to substantial illness, mortality, and an economic burden on health care and society [1,2]. With aging populations and the increased prevalence of chronic diseases, primary care is strained due to a shortage of general practitioners (GPs) and practice nurses (PNs) and an increased workload [3-5]. Remote patient management (RPM) for preventive care is a relatively new approach that can improve reaching blood pressure (BP) targets, aid weight loss, and facilitate lifestyle change [6-10]. However, a significant gap remains in the large-scale implementation of RPM within integrated care [11,12].

In the Netherlands, a matched cohort study of a multicomponent RPM intervention, “cardiovascular risk management (CVRM)-Box,” has been conducted to evaluate the effect of RPM within primary care settings for enhancing BP control, weight, and consultation frequency [13]. Patients at a high risk of major adverse cardiovascular events (MACEs), including myocardial infarction (MI), stroke, or transient ischemic attack, were recruited across 6 primary care practices [13]. Previous implementation studies of the CVRM-Box intervention, guided by the RE-AIM (Reach, Effectiveness, Adoption, Implementation and Maintenance) framework and the Consolidated Framework for Implementation Research, have shown that successful implementation depends on factors such as compatibility with routine practice, perceived workload for health care professionals, technical feasibility, and organizational support [14,15]. In addition to these implementation determinants, the cost-effectiveness of RPM interventions is a key consideration for their adoption, long-term sustainability, and large-scale implementation in routine primary care.

Health economic assessments have traditionally been tailored for pharmacological studies and medical devices and may not adequately capture the multifaceted nature of RPM interventions. Capturing the broader benefits of RPM interventions, such as enhancing health care professional efficiency through increasing patient self-management, is important for informed decision-making [16-18]. Effective implementation and scaling of RPM involves new value propositions shared among different stakeholders and capturing these different values that arise from RPM-supported care [19,20]. Waiting for numerous studies with larger time horizons and sample sizes is impractical and costly, as information is essential for upfront investment to achieve large-scale adoption. More insight into realistic scenarios of upscaling is crucial for informed decision-making [21].

Therefore, this study aimed to model the long-term cost-effectiveness, budget impact, and reduction in required visits to assess the potential scale-up of a multicomponent RPM intervention for the high-risk CVD population in primary care.

## Methods

### Study Design

The current study constitutes a model-based evaluation of CVRM-Box versus care as usual (CaU) [22]. This approach enables the transparent combination of multiple data sources in one modeling framework to estimate long-term health and economic outcomes under each care pathway (Figure 1A). Patients at risk of first or recurrent MACE were simulated throughout their remaining lifetime according to each care pathway. Our model input parameters incorporated data from 3 main sources: (1) primary data from the prospective propensity score-matched CVRM-Box cohort study [13], which informed the intervention effect on systolic blood pressure (SBP) and intervention-related resource use and costs; (2) primary data from the Extramural Leiden University Medical Center (LUMC) Academic Network (ELAN) database [23,24], which informed baseline risk factor profiles and CaU costs for at-risk patients; and (3) published literature, public datasets, and published Dutch costing guidance, which informed calculations of event probabilities, event and postevent costs, and health utilities. Table 1 summarizes key parameters and sources, while Multimedia Appendices 1 and 2 provide full parameterization and transition-probability calculations. Figure 1A presents a general schematic of our study design.

**Figure 1.**
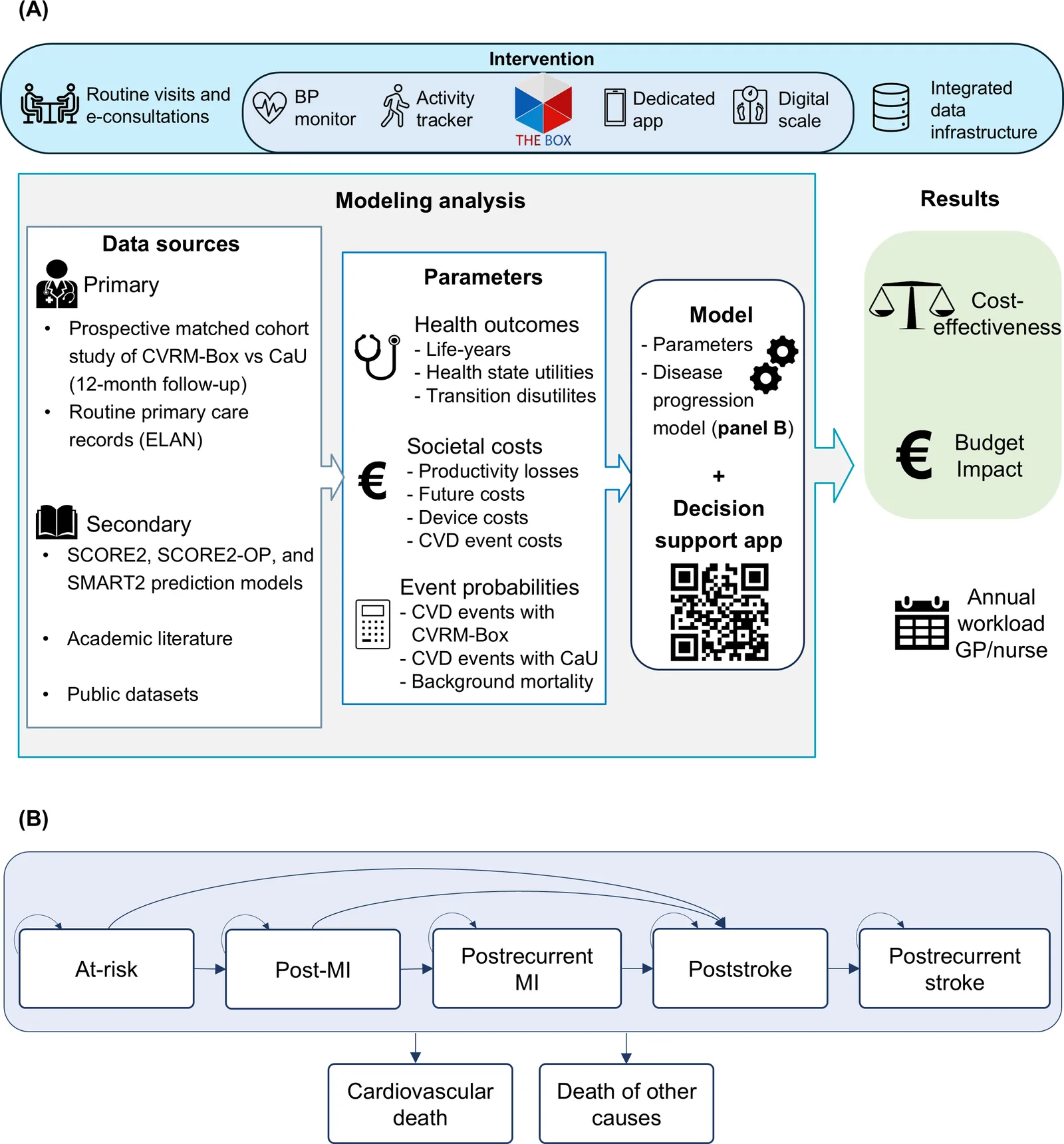
Study design schematic (A) and disease progression model (B). Arrows represent possible transitions between 1-year model cycles. BP: blood pressure; CaU: care as usual; CVD: cardiovascular disease; CVRM: cardiovascular remote management; ELAN: Extramural LUMC Academic Network; GP: general practitioner; MI: myocardial infarction.

**Table 1.** Model input parameters with corresponding credible intervals (CrIs), sources, and selected posterior distributions.

Model input parameter	Estimate (95% CrI)	Distribution^a^	Source
Proportions, %			
Male	53.1	Fixed	[13] CVRM-Box^b^^,^^c^
Subgroups			
Controlled BP^d^, no CVD^e^ history	48.2	Fixed	[13] CVRM-Box^b^^,^^c^
Controlled BP, MI^f^/stroke history	12.4	Fixed	[13] CVRM-Box^b^^,^^c^
Uncontrolled BP, no CVD history	34.5	Fixed	[13] CVRM-Box^b^^,c^
Uncontrolled BP, MI/stroke history	4.9	Fixed	[13] CVRM-Box^b^^,^^c^
Intervention effect			
Reduction in SBP^g^, controlled BP subgroup, mm Hg	1.3 (−1.50 to 4.26)	Normal	[13] CVRM-Box^b^^,^^c^
Reduction in SBP, uncontrolled BP subgroup, mm Hg	3.5 (−0.70 to 7.94)	Normal	[13] CVRM-Box^b^^,^^c^
Smoking cessation proportion	0.112 (0.023 to 0.180)	Triangular	[10]^c^
Costs per patient, €^h^			
Initial cost, intervention			
Device	186.8	Fixed	[13] CVRM-Box^b^^,^^c^
Personnel	26	Fixed	[13] CVRM-Box^b^^,^^c^
State-specific (annual) costs			
At risk with controlled BP, intervention	88 (50 to 141)	Gamma	[13] CVRM-Box^b^^,^^c^
At risk with uncontrolled BP, intervention	94 (53 to 150)	Gamma	[13] CVRM-Box^b^^c^
At risk with controlled BP, CaU^i^	61 (45 to 81)	Gamma	ELAN^c^
At risk with uncontrolled BP, CaU	76 (56 to 101)	Gamma	ELAN^c^
Incremental cost, post-MI^j^	1762 (1473 to 1996)	Triangular	[25,26]^c^
Incremental cost, poststroke^j^	9609 (7897 to 12,084)	Triangular	[27,28]^c^
Incremental cost, recurrent MI^k^	1059 (204 to 1857)	Triangular	[25,26]^c^
Incremental cost, recurrent stroke^k^	1475 (290 to 2514)	Triangular	[27,28]^c^
Transition costs			
MI event	13,929 (7612 to 22,009)	Triangular	[25,26,29-31]^c^
Stroke event	16,606 (8842 to 23,266)	Triangular	[27,28,32-35]^c^
Fatal cardiovascular event	13,929 (7612 to 22,009)	Triangular	Assumed^c^
Productivity loss after MI/stroke	19,860 (18,345 to 21,229)	Triangular	[27,32,36-40]^c^
Utilities and disutilities			
At-risk, controlled BP	0.830 (0.791 to 0.868)	Beta	CVRM-Box^b^^,^^c^
Annual disutility, uncontrolled BP^j^	0.062 (0.031 to 0.098)	Triangular	[41-43]^c^
Annual disutility, post-MI^j^	0.079 (0.023 to 0.141)	Triangular	[41-43]^c^
Annual disutility, poststroke^j^	0.118 (0.052 to 0.201)	Triangular	[41-46]^c^
Annual disutility, recurrent MI^k^	0.080 (0.019 to 0.140)	Triangular	[47,48]^c^
Annual disutility, recurrent stroke^k^	0.063 (0.013 to 0.105)	Triangular	[32,44,46]^c^
Transition disutilities			
MI event	0.111 (0.047 to 0.174)	Triangular	[43,48,49]^c^
Stroke event	0.139 (0.028 to 0.231)	Triangular	[32,44,49]^c^
Stroke event after MI	0.341 (0.291 to 0.388)	Beta	[49]^c^

a Assumed posterior distribution for probabilistic resampling.

b Referring to the matched prospective study of CVRM-Box described in the Introduction.

c Further details on parametrization are shown in Table S1 in Multimedia Appendix 1.

d BP: blood pressure.

e CVD: cardiovascular disease.

f MI: myocardial infarction.

g SBP: systolic BP.

h EUR €1=US $1.11 as of 29 December 2023.

i CaU: care as usual.

j Incremental cost or disutility relative to the at-risk state.

k Incremental cost or disutility relative to the at-risk state and post-MI/poststroke state.

The cost-effectiveness of CVRM-Box was described using incremental cost-effectiveness ratios (ICERs) and cost-effectiveness acceptability curves [50]. We additionally conducted 2 budget impact analyses (BIAs) and estimated annual reductions in workload for GPs and PNs. Reporting followed the CHEERS (Consolidated Health Economic Evaluation Reporting Standards; the completed CHEERS reporting checklist is available as Checklist 1) [51]. Finally, a user-friendly R Shiny app was developed [52] to facilitate interaction with and modifications to the model by decision-makers and researchers, such as assuming different values for key model parameters (eg, higher costs, lower intervention effects) to represent alternative scenarios or assumptions. The web-based app is accessible at jmheij.shinyapps.io/CVRM-Box-model.

### Ethical Considerations

The present study was a model-based secondary analysis and involved no new participant recruitment or data collection. The prospective CVRM-Box study [13], which informed various model input parameters, was approved by the Medical Ethics Committee of Leiden University Medical Center (N21.126), and participants provided written online informed consent. The same committee determined that the ELAN data resource was not subject to the Dutch Medical Research Involving Human Subjects Act (reference G18.070); individual informed consent was therefore waived, and individuals could withdraw through an informed opt-out procedure [24]. Analyses were conducted using deidentified CVRM-Box data and anonymized ELAN data, with access restricted to authorized personnel. Participants in the CVRM-Box study received no financial or other compensation.

### Population and Setting

The modeled cohort represents primary care patients who are part of the Dutch integrated care programs for increased risk or history of CVD. For the main analysis, we assumed the population receives the intervention at age 50. In line with the prospective study of CVRM-Box, the cohort was 53% male and had a moderate to high risk (10%-15%) of 10-year CVD mortality [53] or a history of MI or stroke.

To account for patient and effect heterogeneity, 4 subgroup-specific analyses were conducted by BP status (ie, controlled/uncontrolled) and history of MI or stroke (ie, history/no history). The proportions of patients in each subgroup (Table 1) were consistent with the prospective study of CVRM-Box [13].

### Interventions

#### CVRM-Box

The modeled CVRM-Box intervention reflects the multicomponent RPM program evaluated in van Hattem et al [13], which was a 12-month matched cohort study across 6 Dutch primary care practices. Because the present model is stratified by BP-control status, the BP-effect parameters used here (Table 1) were taken from the subgroup-specific estimates of that study: an adjusted office-measured SBP difference of –1.3 (95% CI –4.1 to 0.5) mm Hg in patients with controlled BP at baseline and –3.5 (95% CI –7.5 to 0.5) mm Hg in patients with uncontrolled BP, neither reaching conventional statistical significance. Despite the modest office-based effect, the intervention significantly increased the proportion of patients achieving controlled BP (<140 mm Hg) over follow-up, and home (CVRM-Box) BP measurements showed a larger reduction than office measurements (SBP –5.5 mm Hg, *P*<.001). The intervention was also associated with statistically significant reductions in body weight (–0.9 kg) and BMI (–0.3 kg/m²) and a lower overall consultation frequency (rate ratio 0.82, *P*=.002). These primary-data estimates, particularly the subgroup-specific SBP differences and the consultation-frequency reduction, directly informed the intervention effect sizes and resource-use parameters used in the present model.

Eligible patients underwent a consultation with an assistant who introduced “The CVRM-Box.” The CVRM-Box was developed within a regional multistakeholder innovation environment, “Gezonde Zorg, Gezonde Regio” [14,54], a multistakeholder initiative involving health care providers and a major regional health insurer. This collaboration included long-term funding agreements, in which efficiency gains were redistributed between providers and the insurer. This structure aimed to align financial incentives and support sustainable implementation of digital care [55]. The intervention included a digital health infrastructure comprising a BP monitor (Wireless Blood Pressure Monitor; Withings), weighing scale (Smart Body Scale Analyzer; Withings), activity tracker (Move, Withings), and a dedicated mobile-based lifestyle app (LUMC Care) integrated in hospital and primary care electronic health records (Medicom, PharmaPartners) (Figure 1A). To facilitate effective use of the CVRM-Box at home, patients were supported by a GP assistant (a student) during dedicated consultations. These sessions included assistance with pairing the monitoring devices to the patient’s smartphone, guidance on navigating the CVRM-Box app, and time to address questions or concerns. Patients were instructed to perform weekly BP and weight measurements and were provided with practical advice via the mobile-based lifestyle app on interpreting their results. In addition, individualized targets for weight and daily steps were defined. Patients were instructed to contact the PN in case of very high BP readings (>180 mm Hg), repeated elevated measurements (>140 mm Hg on 3 consecutive occasions), persistently increased values, or the occurrence of symptoms such as dizziness, headache, or chest pain. A follow-up appointment within 6 weeks was scheduled to resolve potential technical problems. The GP assistant also managed a help desk service that patients could reach for technical issues with their devices.

Measurement results were stored locally on the patient’s smartphone and subsequently transmitted to the servers of the respective app providers (Withings). To safeguard participant privacy, each patient was assigned an @hlc.nl email address consisting of a randomly generated identifier and password, which was used solely for study-related logins. The @hlc.nl domain was managed by the LUMC, and all associated data were stored on secure LUMC servers. Data from Withings devices were retrieved through the Withings Application Programming Interface. The Withings Application Programming Interface enabled automatic transfer of device data into the hospital and GP electronic medical record system using a secure OAuth2 authentication process.

All transmitted measurements were reviewed at least weekly by the PN using a built-in dashboard within the electronic patient record. Because this dashboard was embedded within the existing workflow, PNs did not need to review incoming measurements through a parallel/separate monitoring system, limiting additional cognitive burden and disruption. Automated alerts flagged abnormal values, prompting the nurse to contact patients when needed. Depending on the findings, medication adjustments could be made or follow-up appointments scheduled. If no mobile health measurements were received for 3 consecutive weeks, GP assistants contacted patients by phone to emphasize the importance of continued monitoring. Further details about the CVRM-Box intervention are described elsewhere [13].

#### CaU

CaU was defined as the current CVRM practice within the primary integrated care programs for individuals with increased cardiovascular risk or a history of CVD. This protocolized program focused on lifestyle modifications and the treatment of BP and cholesterol levels and was primarily conducted by the PN [56]. Yearly check-ups at the GP included a local BP measurement and laboratory examination. Most visits (95%) were assumed to be with the PN, while the other visits (5%) took place with the GP when necessary. Thus, routine cardiovascular monitoring in both care pathways was predominantly PN-led, with limited direct GP involvement.

### Model Structure

We developed a cohort Markov model with 1-year cycles to compare CVRM-Box versus CaU over a lifetime horizon. The underlying disease-progression structure (health states and allowed transitions) was identical between arms (Figure 1B), but the disease progression rates differed by arm. Specifically, baseline risk-factor profiles and CaU costs were informed by ELAN routine primary care data [23,24], while CVRM-Box intervention effects on SBP (by BP-control subgroup) and intervention-related costs were sourced from the prospective matched cohort study [13]. Cardiovascular event risks for the transitions shown in Figure 1B were generated using SCORE2/SCORE2-OP [57,58] and SMART2 [59] risk prediction equations populated with subgroup-specific mean risk factors and applying CVRM-Box–related changes in SBP in the intervention arm (more details in Transition probabilities subsection).

The Markov model (Figure 1B) simulated 3 cardiovascular events: nonfatal MI, nonfatal stroke, or fatal cardiovascular event. Additionally, the “Postrecurrent” states were included to account for the greater risk and consequences of recurrent nonfatal events. As the costs and burden of stroke are generally greater than those of MI [25,27,41,49,60], the model does not allow for transitions from stroke to MI states. Finally, patients could experience a fatal cardiovascular event or die of other causes. Patients with no CVD history entered the model in the “At risk” state, while patients with a CVD history entered the model in the post-MI (64%) and poststroke (36%) states, in line with the proportions observed in ELAN data [23,24].

### Model Input Parameters

#### Probabilistic Approach

The main analysis was probabilistic, whereby parameter estimates were resampled 1000 times based on their SEs and assumed posterior distributions [50]. This probabilistic approach enabled us to reflect uncertainty in the available evidence (eg, the uncertainty surrounding key parameters) by running the model many times with plausible alternative input values, so results could be reported as ranges and the probability that CVRM-Box represents good value for money. All model parameters with corresponding sources [10,25-49] and uncertainty ranges are summarized in Table 1 (details in Table S1 of Multimedia Appendix 1).

#### Transition Probabilities

The transitions in Figure 1B were simulated using age- and subgroup-specific transition probability matrices. Transitions from the “At-risk” state to post-MI/stroke or cardiovascular death were parameterized using the SCORE2 and SCORE2-OP prediction equations, including the appropriate recalibration scales for the Netherlands (ie, corresponding to the “low risk” region) [57,58]. Transitions between post-MI/stroke states and from post-MI/stroke to cardiovascular death were parameterized using the SMART2 prediction equations, including the appropriate recalibration ratio for the Netherlands (ie, corresponding to the “low risk” region) [59]. All prediction equations were populated with subgroup-specific mean risk factor values (ie, cholesterol, BP, etc, reported in Tables S2-S7 of Multimedia Appendix 1) obtained from ELAN data [23,24] (representative of CaU). All-cause mortality followed population life tables corrected for CVD-related mortality [61,62]. Transition probability calculations are detailed in Multimedia Appendix 2.

The effect of CVRM-Box was modeled as a reduction in the absolute risk of MACE (ie, lower transition probabilities) resulting from an expected reduction in BP and smoking with the intervention (Table 1). The reduction in BP was equivalent to the adjusted difference in BP estimated in the matched prospective study of CVRM-Box (Table 1). Specifically, the intervention study of CVRM-Box reported an adjusted mean reduction in SBP of −1.3 mm Hg among patients with controlled BP and a −3.5 mm Hg reduction among patients with uncontrolled BP [13]. Smoking cessation was assumed at 13% of patients with a wide credible interval of 2% to 18% [10].

#### Costs

In line with Dutch guidelines for economic evaluations, we followed a societal perspective and discounted costs at 3.5% per yearly cycle [63].

Annual intervention and CaU costs for at-risk patients were estimated from the prospective study of CVRM-Box [13] and ELAN data [23,24], respectively, while the incremental annual costs for nondeath states were sourced from literature (Table 1). The literature sources informing the incremental annual costs for stroke states included a Dutch 2-year analysis of health care use and costs among stroke survivors in the Restore4Stroke Cohort [27], and a German lifetime cost analysis of stroke survivors [28]. For the incremental annual costs in MI states, sources included a 6-year cohort study among MI patients that reported annual health care use [25], and parameters used in a previous Dutch economic evaluation [26]. Nonhealth care costs included work productivity losses (ie, reduced working and/or work leave), estimated using the friction cost method [64] and accrued only while the cohort was younger than 67 years (Table 1). The degree and duration of work productivity loss was informed by ranges reported in various studies, most of which showed that work leave after a nonfatal CVD event lasts approximately 120 days and high proportions of early retirements [27,32,36-40].

Our modeling approach integrated state-specific and transition-specific costs (Table 1). State-specific costs represent the aforementioned annual/recurring costs associated with each health state, while transition-specific costs account for any one-time/short-term costs associated with the cardiovascular events themselves [65]. Existing literature that reported on transition-specific costs, such as initial hospitalization and treatment after a stroke [27,28,32-35] or MI event [25,26,29-31], were used to inform those parameters (Table 1 and Multimedia Appendix 1).

Finally, to avoid overestimating the economic benefits of preventing fatal cardiovascular events, we used the PAID 3.0 tool [63,66] to include future unrelated medical costs attributable to any increases in life expectancy with the intervention. The PAID 3.0 tool was developed to provide standardized, age- and sex-specific estimates of health care expenditures unrelated to the index condition that accrue in additional life-years following (preventive) interventions [63,66]. Incorporating these costs is recommended in economic evaluations to avoid overstating an intervention’s net benefits [63].

#### Outcomes

Total quality-adjusted life years (QALYs) were calculated as the sum of utilities over all cycles, applying a discount rate of 1.5% per cycle [63]. Similarly to costs, our model accounts for state-specific utilities and transition-specific disutilities (all reported in Table 1). State-specific utilities represent the annual utility of patients *excluding* any acute, short-term burden associated with the acute transition events themselves (which were modeled as transition-specific disutilities).

The baseline annual utility of 0.83 for patients in the “At-risk, controlled BP” state was estimated using primary data from the matched prospective CVRM-Box cohort study [13]. From that baseline value, annual disutilities for uncontrolled BP and all postevent (stroke and MI) states were informed by published national catalogs of preference-based utility values across multiple chronic conditions (including CVD) [41-43], evidence from longitudinal studies on cardiovascular populations [32,44,48], population-based health-related quality of life analyses [45,47], and a systematic review/meta-analysis of utility weights in stroke patients [46]. Four of these studies also reported utility values that were used to derive our model’s transition-specific disutility parameter estimates (Table 1) [32,43,44,48], as well as an additional vignette study [49].

### Assumptions

As with every model, ours is subject to simplifying assumptions (details in Multimedia Appendix 2). First, there is no waning in effect nor change in adherence to the intervention, although this is addressed in later scenario analyses. Second, patients can only experience 1 recurrent event of the same type. Although third/fourth events occur in practice, these are much less frequent, and evidence to inform corresponding parameters is lacking. Third, the costs of a fatal event are equivalent to the lowest of the nonfatal events to avoid overestimating the benefit of preventing cardiovascular deaths.

### Model Validation

The Technical Verification (TECH-VER) checklist was used to test our model’s credibility and technical implementation [67]. The checklist consists of white-box and black-box tests designed to identify errors/inconsistencies in model implementation. The completed TECH-VER checklist is reported in Table S8 of Multimedia Appendix 1.

### Uncertainty Analyses

#### Deterministic Sensitivity Analysis

To describe the influence of each model parameter on our results, we performed deterministic sensitivity analyses (DSAs) on all parameters assuming alternative (ie, 25% lower or 25% higher) point estimates.

#### Scenario Analyses

To explore the sensitivity of results to more general assumptions, 4 scenarios were explored: (1) starting age of 40 years, to explore how earlier receipt of the intervention influences cost-effectiveness; (2) the effectiveness of CVRM-Box decreases by 40% after 5 years, reflecting a reasonable reduction in adherence/discontinuation; (3) health care perspective (excluding nonhealth care costs) [63]; and (4) no discounting of costs and utilities [63].

### BIA

To estimate the short- or medium-term budgetary requirements for rolling out CVRM-Box, we conducted 2 BIAs assuming rollout at the regional (South-Holland-North region) and national levels, with a time horizon of 5 years. The methodological approach and assumptions for both BIAs are detailed in Multimedia Appendix 2. Briefly, the South-Holland-North region has 10,256 patients eligible to receive the intervention over 5 years (8344 in the first year) [68]. For the national-level BIA, the corresponding eligible population was estimated at 2,177,350 (with 1,917,812 in the first year) based on publicly available data [69].

### Workload Reduction in Care Delivery

RPM interventions may by design result in lower provider workload, an outcome not completely captured by cost-effectiveness analyses or BIAs. Therefore, we conducted an additional exploratory workload analysis to estimate potential savings in time and resource utilization with the intervention. We estimated the annual reduction/change in (1) time spent on patient contacts (short and long visits) and (2) number of e-consultations per GP and PN in a reference Dutch primary care practice (in Dutch, a “normpraktijk”). A reference primary care practice in the Netherlands has on average 1 GP, 0.83 PNs, and 2100 registered patients [70], of which 420 have a history or high risk of CVD [68].

To estimate the amount of time spent on care by GPs and PNs annually, we used data from the CVRM-Box prospective study [13] about the frequency of e-consultations, short visits, and long visits with GPs and PNs (by subgroups of controlled and uncontrolled BP). These per-patient frequencies were converted to expected practice-level annual volumes by multiplying (for each BP-control subgroup and contact type) the relevant per-patient frequency by the expected number of eligible patients with CVD in a reference primary care practice: 2100 registered patients × 420/2100 patients with CVD × 55% assumed eligible=231 eligible patients per practice. This eligible practice population was then split into controlled and uncontrolled BP according to our main analysis proportions. Annual time requirements (hours) per provider were calculated by multiplying expected annual visit counts by standard scheduled visit durations and dividing by the number of practitioners per reference primary care practice (1 GP; 0.83 PN), while remote consultations were reported as annual counts per provider. Consistent with current practice in the Netherlands, a short visit was calculated to last 10 minutes (with the GP) and 15 minutes (with a PN), while long visits lasted 20 minutes (with a GP) and 30 minutes (with a PN), respectively. The complete formulas and assumptions are provided in Multimedia Appendix 2.

## Results

### Main Analysis

Overall, CVRM-Box was €924 (95% CrI –467 to 2238) more costly and yielded 0.05 (95% CrI –0.02 to 0.12) more QALYs than CaU, resulting in an ICER of €17,340 per QALY gained (Table 2) from a societal perspective (EUR €1=US $1.11 as of 29 December 2023). At the commonly applied willingness-to-pay threshold of €20,000/QALY for prevention in the Netherlands, the overall probability of CVRM-Box being cost-effective over CaU was 60% (Figure 2A).

In terms of subgroup-specific results, CVRM-Box was ≥70% likely to be cost-effective and had ICERs below €20,000/QALY in all subgroups except for patients with controlled BP and no history of MI/stroke (Table 2 and Figure 2A). For the latter subgroup, the higher ICER of €42,384/QALY was mainly explained by the fact that CVRM-Box did not meaningfully influence MACE rates in this group of patients, in which BP levels were already within target range.

**Table 2.** Costs, quality adjusted life years, life expectancy, and incremental cost-effectiveness ratios in base-case and scenario analyses^a^.

Model outcome	Current practice	Cardiovascular remote management–Box
Health care costs (€^b^), mean (95% credible interval)		
Controlled, no history	6218 (4256 to 8431)	7131 (4960 to 9439)
Controlled, history of myocardial infarction (MI)/stroke	90,538 (63,338 to 120,369)	91,548 (64,820 to 121,417)
Uncontrolled, no history	7879 (5369 to 10,730)	8853 (6218 to 11,704)
Uncontrolled, history of MI/stroke	92,542 (64,159 to 123,850)	94,192 (66,664 to 125,263)
Pooled	21,476 (14,964 to 28,656)	22,459 (16,048 to 29,691)
Nonhealth care costs (€), mean (95% credible interval)		
Controlled, no history	603 (557 to 645)	591 (546 to 632)
Controlled, history of MI/stroke	9034 (8345 to 9657)	8870 (8190 to 9501)
Uncontrolled, no history	828 (764 to 885)	791 (727 to 854)
Uncontrolled, history of MI/stroke	11,336 (10,471 to 12,118)	10,947 (10,085 to 11,772)
Pooled	2252 (2080 to 2407)	2194 (2024 to 2353)
Total societal costs (€), mean (95% credible interval)		
Controlled, no history	6821 (4820 to 9076)	7722 (5517 to 10066)
Controlled, history of MI/stroke	99,572 (71,692 to 130,026)	100,418 (73,052 to 130,893)
Uncontrolled, no history	8706 (6144 to 11,615)	9643 (6964 to 12,518)
Uncontrolled, history of MI/stroke	103,878 (74,641 to 135,967)	105,140 (76,492 to 137,096)
Pooled	23,728 (17,038 to 31,066)	24,652 (18,087 to 32,022)
Quality-adjusted life years (QALYs), mean (95% credible interval)		
Controlled, no history	20.66 (19.66 to 21.61)	20.68 (19.68 to 21.63)
Controlled, history of MI/stroke	12.28 (10.34 to 13.98)	12.38 (10.41 to 14.12)
Uncontrolled, no history	18.74 (17.38 to 19.98)	18.79 (17.42 to 20.01)
Uncontrolled, history of MI/stroke	9.86 (7.56 to 11.89)	10.07 (7.74 to 12.15)
Pooled	18.43 (17.3 to 19.52)	18.48 (17.34 to 19.58)
Modeled life-expectancy in years, mean (95% credibel interval)		
Controlled, no history	32.16^c^	32.2 (32.13 to 32.26)
Controlled, history of MI/stroke	21.45	21.62 (21.31 to 21.94)
Uncontrolled, no history	31.45	31.57 (31.44 to 31.69)
Uncontrolled, history of MI/stroke	18.78	19.21 (18.73 to 19.7)
Pooled	29.93	30.03 (29.89 to 30.17)
Incremental cost-effectiveness ratio		
Controlled, no history	—^c^	€42,384/QALY
Controlled, history of MI/stroke	—	€8806/QALY
Uncontrolled, no history	—	€15,708/QALY
Uncontrolled, history of MI/stroke	—	€5849/QALY
Pooled	—	€17,340/QALY
Scenario analysis incremental cost-effectiveness ratios, pooled results		
Health care perspective	—	€18,434/QALY
No discounting	—	€26,882/QALY
Starting age of 40 years	—	€15,087/QALY
Intervention effect reduces by 40% after 5 years	—	€23,110/QALY

a Costs and quality-adjusted life years are per patient lifetime in the population.

b EUR €1=US $1.11 as of 29 December 2023.

c Mean or 95% credible interval not applicable.

**Figure 2.**
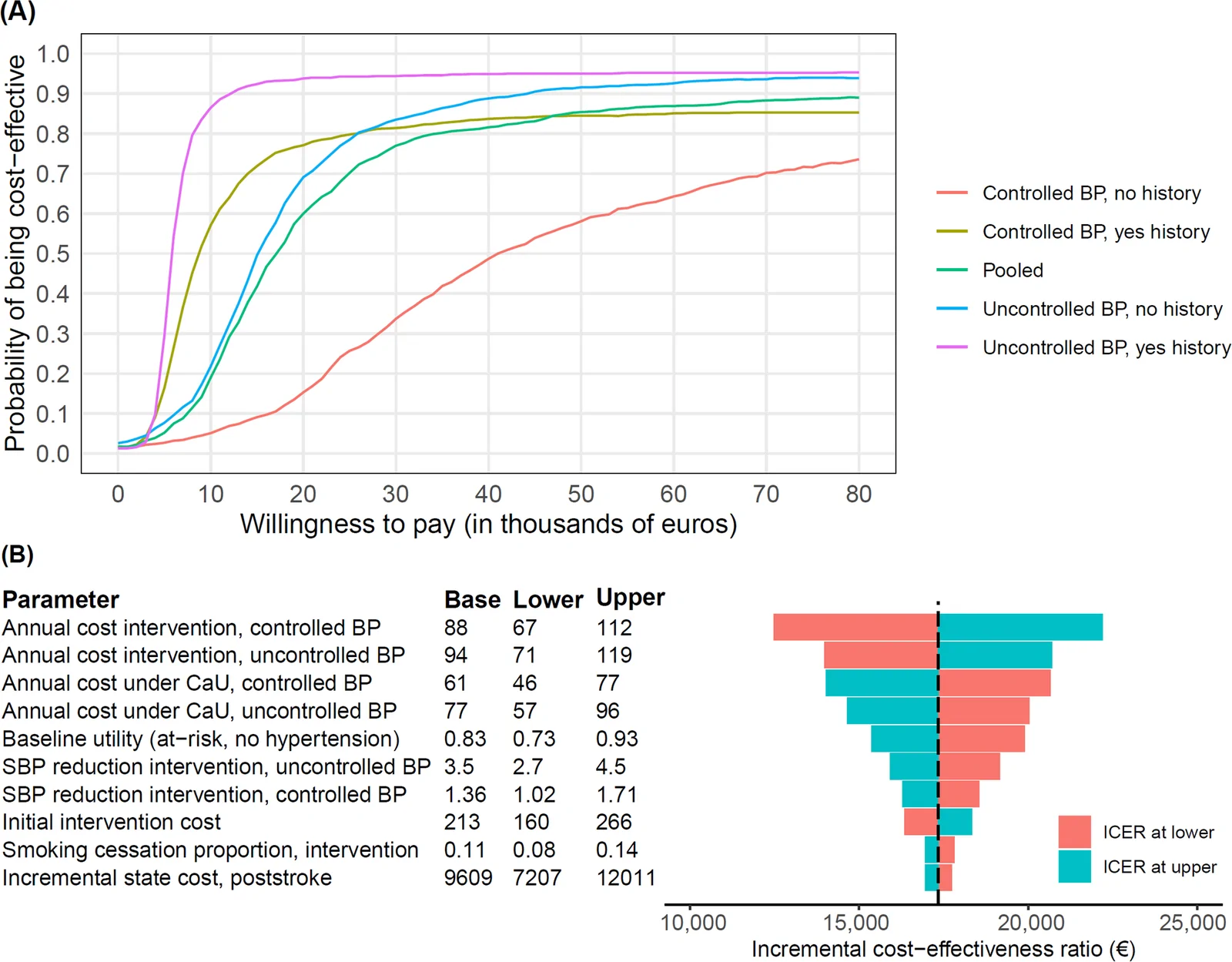
Cost-effectiveness acceptability curves (A) and deterministic sensitivity analysis results (B). (A) The curves represent the probability that CVRM-Box is cost-effective compared to CaU for each willingness to pay on the x-axis. (B) The “Base” values are the parameter values used in the main analysis (Table 1), while the “Lower” and “Upper” values are plausible alternative values. BP: blood pressure (systolic); CaU: care as usual; ICER: incremental cost-effectiveness ratio; SBP: systolic blood pressure.

### DSA

The most influential model parameters were the annual costs of CVRM-Box and CaU (Figure 2B). The model’s sensitivity to these parameters was largely due to the fact that QALY gains with the intervention were small, making differences in costs more influential (especially costs that apply to the entire cohort). All other parameters had a much lower influence on the estimated ICERs.

### Scenario Analyses

The scenario assuming no discounting and the scenario assuming a waning effect of the intervention resulted in ICERs above €20,000/QALY, while a starting age of 40 decreased the overall ICER to €15,087/QALY (Table 2). Subgroup-specific scenario analysis results (Table S9 of Multimedia Appendix 1) showed that the ICERs for CVRM-Box were consistently below €20,000/QALY, with few exceptions in the subgroups with no history of MI/stroke, in line with our main analysis.

### Budget Impact

The pooled budget impact of CVRM-Box was €2.9 million assuming regional rollout (10,256 patients) and €662.2 million assuming national rollout (1,917,812 patients) over 5 years. Nearly 60% of the overall budget impact was attributable to patients with controlled BP and no history of MI/stroke (Figure S1 of Multimedia Appendix 1), due to the fact that the higher intervention costs were not compensated by the (few) prevented events in this subgroup. Conversely, the budget impact was much lower in other subgroups, ranging from €0.26 to €1.01 million at the regional level and €59.3 to €232.5 million at the national level (Figure S1 of Multimedia Appendix 1). This was especially the case in the subgroup with uncontrolled BP and a history of MI/stroke, where the 5-year budget impact was slightly negative.

### Workload Reduction

Assuming a reference Dutch primary care practice, CVRM-Box would be expected to require more time spent on long visits with patients and less time spent on short visits each year (Table 3). The reduction in time spent on short visits compensated for the additional workload on long visits, resulting in a net annual reduction in workload that was substantial for PNs but negligible for GPs. Annually, PNs would spend 25.9 fewer hours on patient contacts and handle 44.7 fewer remote consultations, whereas the corresponding change for GPs was small (0.5 h and 3.3 fewer remote consultations). The greatest workload reductions were among PNs treating patients with uncontrolled BP (Table S10 of Multimedia Appendix 1).

**Table 3.** Annual expected reductions in workload with the intervention, per general practitioner and practice nurse in a reference Dutch primary care practice^a^.

Care activity	CVRM^b^-Box, annual workload	Care as usual, annual workload	Annual increase (+) or reduction (−) with intervention
Per practice nurse			
Long patient contacts^c^	157.7 hours	147.2 hours	+10.5 hours
Short patient contacts^d^	94.7 hours	131.1 hours	–36.4 hours
All patient contacts (sum)	252.4 hours	278.3 hours	–25.9 hours
Remote consultations	167.0 consultations	211.7 consultations	–44.7 consultations
Per general practitioner			
Long patient contacts^c^	4.6 hours	4.2 hours	+0.4 hours
Short patient contacts^d^	2.8 hours	3.7 hours	–0.9 hours
All patient contacts (sum)	7.4 hours	7.9 hours	–0.5 hours
Remote consultations	6.9 consultations	10.2 consultations	–3.3 consultations

a “Hours” and “consultations” refer to annual amounts per general practitioner or practice nurse in a reference primary care practice assuming an eligible cardiovascular population of N=231 patients per practice (Multimedia Appendix 2).

b CVRM: cardiovascular remote management.

c Duration of 20 minutes with general practitioner and 30 minutes with practice nurse.

d Duration of 10 minutes with general practitioner and 15 minutes with practice nurse.

## Discussion

### Principal Findings

In this study, the long-term cost-effectiveness, budget impact, and workload reductions associated with the CVRM-Box implemented in primary care were assessed using a model-based evaluation. The CVRM-Box was highly likely to be cost-effective among patients with uncontrolled BP and/or a history of CVD, but not in patients with controlled BP and no history of CVD. Our BIA showed a similar pattern, where most of the incremental costs were attributable to this healthier subgroup. Together, these findings support considering the CVRM-Box primarily for high-risk subgroups, whereas broader adoption for lower-risk patients would require significant financial investment with insufficient QALY gains. Another key finding was that the CVRM-Box was expected to reduce the annual workload of PNs, who deliver the large majority of routine cardiovascular contacts in Dutch primary care. The corresponding change in GP workload was negligible, and because it is of a similar order to the one-off training and set-up required to adopt the intervention, we do not interpret it as a saving in GP time; the workload benefit is therefore essentially a PN benefit, which is where workforce pressure in Dutch primary care is greatest.

Our results were generally robust to extensive uncertainty analyses. Our DSA showed that the annual intervention costs were highly influential, suggesting that reducing the intervention costs would yield the greatest increase in cost-effectiveness. Our scenario analyses showed that delivering the intervention at a younger age (ie, 40 instead of 50 y) would improve cost-effectiveness, and that a 40% reduction in effectiveness/adherence after 5 years would not compromise cost-effectiveness in the 3 highest-risk subgroups (Table S9 of Multimedia Appendix 1).

### Comparison With the Literature

Model-based evaluations of RPM interventions for CVD vary in methodology, making comparisons difficult. However, most studies—including ours—show cost-effectiveness for managing hypertension and long-term CVD [71-73]. In line with this, an economic evaluation of home-based cardiac telerehabilitation reported a high probability of cost-effectiveness despite nonsignificant differences in clinical outcomes and costs [74]. The BP reductions underlying our analysis are likewise consistent with this evidence base. Telemonitoring in the TASMINH4 trial lowered systolic pressure by 4.7 mm Hg relative to usual care [10], and comparable reductions are reported in an individual-patient-data meta-analysis of self-monitoring [75]. Reductions of this magnitude remain clinically relevant at the population level, as an approximately 5 mm Hg systolic reduction has been associated with around a 10% lower risk of major cardiovascular events in a large individual-participant-data meta-analysis of BP-lowering treatment [76], corresponding to a meaningful absolute reduction in events for a scalable primary care population. Our findings contribute to ongoing discussions about the cost-effectiveness assessment processes for digital health interventions. In recent years, the focus has shifted from solely clinical outcomes and cost-effectiveness as value indicators to incorporating clinical workflows and health care professionals’ perspectives [14,77,78]. As also found in our previous implementation studies of the CVRM-Box [14,15], stakeholders suggest that RPM’s impact on health care professional workload is increasingly important, particularly in the context of labor shortages in primary care [14]. Despite this, current practices still prioritize (cost-)effectiveness and BIAs, while considerations related to workload reductions have not yet become mainstream. These indicators are high on the political agenda and are important to decision-makers as factors for the value of RPM in practice. We partly addressed this knowledge gap through our estimation of expected reductions in annual workload for GPs and PNs assuming scale-up in Dutch primary care centers. That said, our workload estimates should be interpreted as exploratory, as they are based on observed consultation frequencies from the prospective CVRM-Box study translated to a Dutch reference practice, and are not embedded within the state-transition model.

Importantly, scaling potential is not determined by economic considerations alone (eg, cost-effectiveness and budget impact). Our previous implementation studies identified additional practical factors influencing large-scale adoption [14,15]. Key barriers included additional clinical and technical tasks for health care professionals and uncertainties regarding reimbursement arrangements, while facilitators included early detection of at-risk patients, the presence of an implementation manager overseeing procedures and implementation metrics, and ambassador roles supporting adoption. Together, these findings reflect that economic evidence should be complemented by attention to practical implementation challenges when considering large-scale deployment of RPM interventions.

In a previous BIA study [79] where upscaling scenarios were calculated from one hospital ward to all Dutch hospitals, it was found that replacing in-hospital care with virtual care did not directly lead to cost savings within a single hospital ward due to labor-intensive virtual care services. The study suggested that minimal involvement of health care professionals, as applied in our intervention, was necessary. Similar to our findings, the previous study noted that scaling regionally (whole hospital) or nationally (all Dutch hospitals) resulted in cost savings relatively quickly. However, they observed cost savings for all patient groups, while we estimated potential cost savings exclusively in the subgroup with uncontrolled hypertension and a history of CVD. This discrepancy is likely due to differences in patient populations.

### Strengths and Limitations

Economic evaluations always rely on assumptions, and despite our validation efforts using the TECH-VER checklist [67], several limitations remain. First, only 3 cardiovascular events were considered. Our choice of events, despite excluding other events like heart failure and sudden cardiac arrest, enabled us to simulate patient transitions using the state-of-the-art SCORE2 and SMART2 risk prediction models, which also focus on MI, stroke, and cardiovascular death. Importantly, long-term cardiovascular outcomes in our model were estimated indirectly through the SCORE2 and SMART2 risk prediction equations (rather than observed cardiovascular events), adding uncertainty to the modeled event rates. Second, the prospective CVRM-Box study had an effect on BMI, but we were not able to account for it due to lacking risk prediction models that include BMI as a predictor. This is a minor limitation, however, as reductions in BMI and BP are closely related, and including an independent effect on BMI would have only resulted in lower ICERs. Third, our exploratory analysis of workload reduction was subject to a higher degree of uncertainty than our main model-based analysis, as it did not use the state-transition model due to limited information about (changes in) consultation frequency per disease state. Nonetheless, even our indicative workload estimates are important for decision-makers given the current lack of data and literature on this question. Future studies should prioritize data collection on workload that can be incorporated into state-transition models to obtain more robust results. Relatedly, the estimated workload reductions should be interpreted as potential reductions in routine patient-contact time annually rather than net implementation time savings, as one-off implementation activities (eg, short training for GPs and PNs and ad hoc troubleshooting) were excluded from these calculations. As these implementation-related time investments apply to the practice population as a whole, their contribution to average annual workload per patient is expected to be negligible. Finally, importantly, our model did not account for transitions from uncontrolled to controlled BP status, potentially leading to more conservative estimates of cost-effectiveness. A microsimulation would address these challenges more effectively and allow for unique patient trajectories to be modeled.

A further consideration concerns the evidence on the intervention effect itself. The office-measured systolic reduction did not reach conventional statistical significance and was derived from a matched, controlled pre-post study rather than a randomized trial. Because the main analysis is probabilistic, the uncertainty in this estimate, including its CI, is carried through to the incremental cost and QALY distributions, which is why our cost-effectiveness conclusions are restricted to the higher-risk subgroups. A controlled pre-post design with broad inclusion criteria, such as that of the source prospective study [13], may also reflect the real-world effect more closely than a selective trial, and the proof of concept for home BP monitoring is already established in randomized trials. A placebo or regression-to-the-mean component in the home measurements, which lacked a control group, cannot be fully excluded, although these measurements avoid the well-known white-coat response [80,81] and the economic analysis relies on the office estimates that were matched to a control group.

Despite these limitations, our study has several strengths. A significant strength is our use of primary data from the matched cohort study of CVRM-Box to inform key model parameters. Other key parameters, including risk factor data for the SCORE2 and SMART2 risk prediction models, came from primary data from the comprehensive ELAN database, which is representative of the current primary care CVD population in a large Dutch region [24]. Our extensive sensitivity and scenario analyses transparently reflect the influence of key assumptions and uncertainties on results, strengthening our overall conclusions.

### Implications for Future Research and Practice

This study highlights the importance of incorporating diverse value indicators (ie, cost-effectiveness, budget impact, and workload) into the evaluation of digital health interventions. Payers often rely on payment- and results-based models to align incentives among payers, providers, and patients [77]. By integrating workload for health care professionals into the cost-effectiveness framework, our study offers a more comprehensive perspective to aid decision-making. Workload reductions may substantially influence resource allocation and reimbursement decisions for RPM interventions, particularly in the context of an increasingly constrained primary care workforce. However, real-world implementation decisions should also account for contextual factors not explicitly modeled here, including local staffing capacity, digital infrastructure and support requirements (eg, device logistics, interoperability with electronic health records), and equity considerations. Notably, provider incentive structures may also influence adoption and generalizability across health care systems. In systems with substantial capitation components, including Dutch primary care, the revenue impact of reduced in-person consultations is attenuated relative to predominantly fee-for-service settings, where additional incentive arrangements may be needed to support uptake. Beyond these payment arrangements, several further features of the Dutch setting shape how our findings should be read elsewhere. CVRM here is delivered through protocolized, integrated primary care programs that are largely PN-led, as described above, and is assessed against a relatively explicit national willingness-to-pay threshold for prevention; the intervention itself was financed through the regional shared-savings arrangement outlined in the *Methods*. Where care is instead more physician-led or fee-for-service, where a different cost-effectiveness threshold applies, or where such shared-savings arrangements are absent, the cost, workload, and adoption implications of CVRM-Box may differ appreciably even when its clinical effect is similar. The relative cost-effectiveness ranking across risk subgroups is therefore likely to transfer more readily than the absolute budget-impact and workload estimates, which are the most context-dependent and would need to be reestimated locally. Finally, future research should incorporate a wider range of value indicators focusing on key aspects such as provider workload, and should consider wider factors that may affect implementation.

### Conclusion

This model-based evaluation of CVRM-Box found that the intervention is likely to be cost-effective in high-risk groups (uncontrolled hypertension or a history of CVD) and may reduce workload for health care professionals, while results are less favorable in lower-risk patients. In light of these findings, our findings support considering implementation in primary care, particularly for higher-risk subgroups. Regional considerations and subgroup-specific outcomes should be considered alongside other key factors such as local capacity or infrastructure and equity in decisions about the introduction and implementation of similar interventions. Importantly, because digital health interventions interact closely with the local health system, our findings should be interpreted primarily within the Dutch primary care context and may not directly generalize to health care systems with different organization, prices, or incentives.

## Supplementary material

10.2196/79512 Multimedia Appendix 1 Tables S1 to Table S10, and Figure S1.

10.2196/79512 Multimedia Appendix 2 Methodological details.

10.2196/79512 Checklist 1 CHEERS reporting checklist.

## Abbreviations

BIA: budget impact analysis
BP: blood pressure
CaU: care as usual
CHEERS: Consolidated Health Economic Evaluation Reporting Standards
CVD: cardiovascular disease
CVRM: cardiovascular remote management
DSA: deterministic sensitivity analysis
ELAN: Extramural LUMC Academic Network
GP: general practitioner
ICER: incremental cost-effectiveness ratio
LUMC: Leiden University Medical Center
MACE: major adverse cardiovascular event
MI: myocardial infarction
PN: practice nurse
QALY: quality-adjusted life year
RE-AIM: Reach, Effectiveness, Adoption, Implementation, and Maintenance
RPM: remote patient management
SBP: systolic blood pressure
TECH-VER: Technical Verification
