# Supplementary material for: A Cardiovascular Care Pathway Supported by Remote Patient Management in Dutch Primary Care: Cost-Effectiveness, Budget Impact, and Workload Analysis
Source: JMIR Mhealth Uhealth. 2026 Sep 28;14:e79512. doi: 10.2196/79512 (PMC13618991; doi:10.2196/79512)
Supplement: Supplementary file 1 [file mhealth-v14-e79512-s001.docx]

**Contents**

[Table S1. Model input parameters with further details	2](#_Toc201577966)

[Table S2. Mean risk factor values used as predictors in SCORE2 and SCORE2-OP models, for the subgroup with controlled hypertension	5](#_Toc201577967)

[Table S3. Mean risk factor values used as predictors in SCORE2 and SCORE2-OP models, for the subgroup with uncontrolled hypertension	5](#_Toc201577968)

[Table S4. Mean risk factor values used as predictors in SMART2 model, for the subgroup with uncontrolled hypertension entering the model with a history of MI	6](#_Toc201577969)

[Table S5. Mean risk factor values used as predictors in SMART2 model, for the subgroup with controlled hypertension entering the model with a history of MI	7](#_Toc201577970)

[Table S6. Mean risk factor values used as predictors in SMART2 model, for the subgroup with controlled hypertension entering the model with a history of Stroke	8](#_Toc201577971)

[Table S7. Mean risk factor values used as predictors in SMART2 model, for the subgroup with uncontrolled hypertension entering the model with a history of Stroke	9](#_Toc201577972)

[Table S8. Completed TECH-VER checklist of white-and black-box tests	10](#_Toc201577973)

[Table S9. Scenario analysis results. Costs, QALYs, and incremental cost-effectiveness ratios (ICERs) vs care as usual.	15](#_Toc201577974)

[Table S10. Annual expected reductions in workload with the intervention, per GP and PN and by subgroups of controlled and uncontrolled BP.	17](#_Toc201577975)

[Figure S1. Budget impact analysis results assuming scale-up at regional and national levels	18](#_Toc201577976)

**Table S1. Model input parameters with further details**

| **Parameter** | **Estimate (95% CrI)** | **Distribution^a^** | **Source** | **Details, when applicable** |
| --- | --- | --- | --- | --- |
| **Proportions** |  |  |  |  |
| Male | 53.1% | Fixed | [13] CVRM-Box | Equivalent to % male in prospective study of CVRM-Box. |
| Subgroups |  |  |  |  |
| Controlled BP, no CVD history (CN) | 48.2% | Fixed | [13] CVRM-Box | Equivalent to % in prospective study of CVRM-Box. |
| Controlled BP, MI/stroke history (CY) | 12.4% | Fixed | [13] CVRM-Box | Equivalent to % in prospective study of CVRM-Box. |
| Uncontrolled BP, no CVD history (UN) | 34.5% | Fixed | [13] CVRM-Box | Equivalent to % in prospective study of CVRM-Box. |
| Uncontrolled BP, MI/stroke history (UY) | 4.9% | Fixed | [13] CVRM-Box | Equivalent to % in prospective study of CVRM-Box. |
| **Intervention effect** |  |  |  |  |
| Reduction in SBP, controlled BP subgroup | 1.3 mmHg (-1.50, 4.26) | Normal | [13] CVRM-Box | The adjusted difference in BP between intervention and (matched) CaU patients in the prospective study of CVRM-Box after 12 months was 1.3mmHg (SE=1.4). Adjustment was on: practice, measurements from the same participants, sex, baseline blood pressure, age, and a history of CVD. |
| Reduction in SBP, uncontrolled BP subgroup | 3.5 mmHg (-0.70, 7.94) | Normal | [13] CVRM-Box | The adjusted difference in BP between intervention and (matched) CaU patients in the prospective study of CVRM-Box after 12 months was 3.5mmHg (SE=2.1). Adjustment was on: practice, measurements from the same participants, sex, baseline blood pressure, age, and a history of CVD. |
| Smoking cessation proportion | 0.112 (0.023, 0.180) | Triangular | [10] | Mode, minimum, and maximum values of the triangular distribution equal to 0.13, 0, and 0.2 respectively, in line with the referenced study. |
| **Costs** |  |  |  |  |
| Initial cost, intervention |  |  |  |  |
| Device | € 186.8 | Fixed | [13] CVRM-Box | €155.7 for device (actual costs in prospective study) + €31.1 shipping. |
| Personnel | € 26 | Fixed | [13] CVRM-Box |  |
| Annual costs |  |  |  |  |
| At risk with controlled BP, intervention | 88 (50, 141) | Gamma | [13] CVRM-Box | Includes costs of consultations with healthcare providers (GPs and PNs) at € 56.9, costs of the medical service center at € 26, and IT infrastructure costs to send readings to GP, at € 5.1. Standard error assumed to be 25% of the mean |
| At risk with uncontrolled BP, intervention | 94 (53, 150) | Gamma | [13] CVRM-Box | Includes costs of consultations with healthcare providers (GPs and PNs) at € 62.7, costs of the medical service center at € 26, and IT infrastructure costs to send readings to GP, at € 5.1. Standard error assumed to be 25% of the mean |
| At risk with controlled BP, CaU | 61 (45, 81) | Gamma | ELAN | Includes costs of consultations with healthcare providers (GPs and PNs). Standard error assumed to be 15% of the mean |
| At risk with uncontrolled BP, CaU | 76 (56, 101) | Gamma | ELAN | Includes costs of consultations with healthcare providers (GPs and PNs). Standard error assumed to be 15% of the mean |
| Incremental cost, Post-MI | 1762 (1473, 1996) | Triangular | [39,40] | Mode, minimum, and maximum values of the triangular distribution equal to 1800, 1400, and 2069 respectively, drawn from lowest/highest estimates found in the referenced literature. |
| Incremental cost, Post-Stroke | 9609 (7897, 12084) | Triangular | [35,41] | Mode, minimum, and maximum values of the triangular distribution equal to 8000, 7731, and 12959 respectively, drawn from lowest/highest estimates found in the referenced literature. |
| Incremental cost, recurrent MI | 1059 (204, 1857) | Triangular | [39,40] | Mode, minimum, and maximum values of the triangular distribution equal to 1000, 0, and 2122 respectively, drawn from lowest/highest estimates found in the referenced literature. |
| Incremental cost, recurrent stroke | 1475 (290, 2514) | Triangular | [35,41] | Mode, minimum, and maximum values of the triangular distribution equal to 1500, 0, and 2851 respectively, drawn from lowest/highest estimates found in the referenced literature. |
| Transition costs |  |  |  |  |
| MI event | 13929 (7612, 22009) | Triangular | [39,40,42–44] | Mode, minimum, and maximum values of the triangular distribution equal to 10000, 6484, and 24847 respectively, drawn from lowest/highest estimates found in the referenced literature. Annual costs were subtracted from transition costs to avoid double counting. |
| Stroke event | 16606 (8842, 23266) | Triangular | [35,41,45–48] | Mode, minimum, and maximum values of the triangular distribution equal to 17000, 6928, and 25408 respectively, drawn from lowest/highest estimates found in the referenced literature. Annual costs were subtracted from transition costs to avoid double counting. |
| CVD death event | 13929 (7612, 22009) | Triangular | assumed | Assumed equal to the lowest cost of the non-fatal events (i.e., MI) to avoid overestimation of prevention benefit. |
| Productivity loss after MI/stroke | 19860 (18345, 21229) | Triangular | [35,45,49–53] | Mode, minimum, and maximum values of the triangular distribution equal to 19829, 17978, and 21679 respectively, drawn from lowest/highest estimates found in the referenced literature. This also includes information on early retirement due to CVD event. |
| **Utilities** |  |  |  |  |
| At-risk, controlled BP | 0.830 (0.791, 0.868) | Beta | [13] CVRM-Box | As measured at baseline using the EQ-5D-5L and corresponding Dutch utility tariffs [79]. |
| Annual dis-utility, uncontrolled BP | 0.062 (0.031, 0.098) | Triangular | [37,54,55] | Mode, minimum, and maximum values of the triangular distribution equal to 0.05, 0.0246, and 0.11 respectively, drawn from lowest/highest estimates found in the referenced literature. |
| Annual dis-utility, Post-MI | 0.079 (0.023, 0.141) | Triangular | [37,54,55] | Mode, minimum, and maximum values of the triangular distribution equal to 0.06, 0.011, and 0.163 respectively, drawn from lowest/highest estimates found in the referenced literature. |
| Annual dis-utility, Post-Stroke | 0.118 (0.052, 0.201) | Triangular | [37,54–58] | Mode, minimum, and maximum values of the triangular distribution equal to 0.08, 0.04, and 0.23 respectively, drawn from lowest/highest estimates found in the referenced literature. |
| Annual dis-utility, recurrent MI | 0.080 (0.019, 0.140) | Triangular | [59,60] | Mode, minimum, and maximum values of the triangular distribution equal to 0.07, 0.0048, and 0.16 respectively, drawn from lowest/highest estimates found in the referenced literature. |
| Annual dis-utility, recurrent Stroke | 0.063 (0.013, 0.105) | Triangular | [45,56,58] | Mode, minimum, and maximum values of the triangular distribution equal to 0.068, 0.00, and 0.118 respectively, drawn from lowest/highest estimates found in the referenced literature. |
| Transition dis-utilities |  |  |  |  |
| MI event | 0.111 (0.047, 0.174) | Triangular | [38,55,60] | Mode, minimum, and maximum values of the triangular distribution equal to 0.1, 0.0325, and 0.196 respectively, drawn from lowest/highest estimates found in the referenced literature. Annual dis-utility of MI states were subtracted from this transition dis-utility to avoid double counting. |
| Stroke event | 0.139 (0.028, 0.231) | Triangular | [38,45,56] | Mode, minimum, and maximum values of the triangular distribution equal to 0.15, 0.0, and 0.26 respectively, drawn from lowest/highest estimates found in the referenced literature. Annual dis-utility of stroke states were subtracted from this transition dis-utility to avoid double counting. |
| Stroke event after MI | 0.341 (0.291, 0.388) | Beta | [38] | Vignette study assuming a 1-year time horizon for time-trade-off task. Annual dis-utility of stroke and MI states were subtracted from this transition dis-utility to avoid double counting. |

**Table S2. Mean risk factor values used as predictors in SCORE2 and SCORE2-OP models, for the subgroup with controlled hypertension**

|  | **Males aged 40-69 (n=10462)** | | **Females aged 40-69 (n=14314)** | | **Males aged >69 (5770)** | | **Females aged >69 (7398)** | |
| --- | --- | --- | --- | --- | --- | --- | --- | --- |
| **Predictor** | **Mean (n)** | **Proportion (n)** | **Mean** | **Proportion** | **Mean** | **Proportion** | **Mean** | **Proportion** |
| Age, years | 57.8 (10462) | - | 57.0 (14314) | - | 77 (5770) | - | 77.9 (7398) | - |
| Current smoking | - | 0.1 (1052) | - | 0.08 (1114) | - | 0.06 (324) | - | 0.05 (344) |
| Diabetes Mellitus | - | 0.23 (2438) | - | 0.2 (2374) | - | 0.27 (1546) | - | 0.21 (1543) |
| Total cholesterol | 4.9 (8075) | - | 5.3 (11022) | - | 4.45 (4260) | - | 5.1 (5736) | - |
| HDL cholesterol | 1.2 (8050) | - | 1.5 (10825) | - | 1.29 (4222) | - | 1.55 (5607) | - |
| Systolic blood pressure | 126.0 (10462) | - | 124.2 (14314) | - | 127.1 (5770) | - | 127.3 (7398) | - |

**Table S3. Mean risk factor values used as predictors in SCORE2 and SCORE2-OP models, for the subgroup with uncontrolled hypertension**

|  | **Males aged 40-69 (n=14195)** | | **Females aged 40-69 (n=14616)** | | **Males aged >69 (7886)** | | **Females aged >69 (12389)** | |
| --- | --- | --- | --- | --- | --- | --- | --- | --- |
| **Predictor** | **Mean (n)** | **Proportion (n)** | **Mean** | **Proportion** | **Mean** | **Proportion** | **Mean** | **Proportion** |
| Age, years | 57.9 (14195) | - | 57.9 (14616) | - | 77 (7886) | - | 78.5 (12389) | - |
| Current smoking | - | 0.1 (1376) | - | 0.08 (1157) | - | 0.05 (417) | - | 0.04 (473) |
| Diabetes Mellitus | - | 0.19 (2737) | - | 0.14 (2081) | - | 0.25 (1992) | - | 0.2 (2435) |
| Total cholesterol | 5.1 (10934) | - | 5.4 (11657) | - | 4.66 (5813) | - | 5.28 (9502) | - |
| HDL cholesterol | 1.2 (10879) | - | 1.5 (11449) | - | 1.31 (5750 | - | 1.57 (9275) | - |
| Systolic blood pressure | 152.1 (14195) | - | 151.5 (14616) | - | 153.4 (7886) | - | 154.8 (12389) | - |

**Table S4. Mean risk factor values used as predictors in SMART2 model, for the subgroup with uncontrolled hypertension entering the model with a history of MI**

|  | **Age 40-69**  **Males (n= 1126)** | | **Age 40-69**  **Females (n= 438)** | | **Age 70+**  **Males (n= 1708)** | | **Age 70+**  **Females (n= 1279)** | |
| --- | --- | --- | --- | --- | --- | --- | --- | --- |
| **Predictor** | **Mean** | **Proportion** | **Mean** | **Proportion** | **Mean** | **Proportion** | **Mean** | **Proportion** |
| Abdominal aortic aneurysm | - | 0 | - | 0 | - | 0 | - | 0 |
| Age, years | 61.3 (1126) | - | 61.9 (438) | - | 64.3 (1708) | - | 81.1 (1279) | - |
| Aspirin or equivalent | - | 0.84 (1126) | - | 0.75 (438) | - | 0.85 (1708) | - | 0.8 (1279) |
| Coronary artery disease | - | 1 | - | 1 | - | 1 | - | 1 |
| eGFR (CKDEPI) | 80.7 (962) | - | 78.24 (386) | - | 64.3 (1487) | - | 62.9 (1279) | - |
| hs-CRP |  | - |  | - |  | - |  | - |
| Current smoking | - | 0.14 (726) | - | 0.14 (265) | - | 0.06 (1129) | - | 0.04 (795) |
| Cerebrovascular disease | - | 0 | - | 0 | - | 0 | - | 0 |
| Diabetes Mellitus | - | 0.27 (1126) | - | 0.23 (438) | - | 0.30 (1708) | - | 0.27 (1279) |
| Non HDL-cholesterol | 1.15 (915) | - | 1.42 (349) | - | 1.23 (1708) | - | 1.47 (980) | - |
| Peripherial artery disease | - | 0.05 (1126) | - | 0.03 (438) | - | 0.06 (1708) | - | 0.06 (1279) |
| Systolic blood pressure | 147.7 (1126) | - | 148.0 (438) | - | 150.3 (1708) | - | 151.9 (1279) | - |
| Years since first ASCVD diagnosis | 8.4 (1126) | - | 7.1 (438) | - | 12.6 (1706) | - | 10.1 (1279) | - |

**Table S5. Mean risk factor values used as predictors in SMART2 model, for the subgroup with controlled hypertension entering the model with a history of MI**

|  | **Age 40-69**  **Males (n= 1686)** | | **Age 40-69**  **Females (n= 726)** | | **Age 70+**  **Males (n= 2039)** | | **Age 70+**  **Females (n= 1154)** | |
| --- | --- | --- | --- | --- | --- | --- | --- | --- |
| **Predictor** | **Mean** | **Prop.** | **Mean** | **Prop.** | **Mean** | **Prop.** | **Mean** | **Prop.** |
| Abdominal aortic aneurysm | - | 0 | - | 0 | - | 0 | - | 0 |
| Age, years | 60.9 (1686) | - | 61.3 (726) | - | 78.3 (2039) | - | 80.2 (1154) | - |
| Aspirin or equivalent | - | 0.86 (1686) | - | 0.8 (726) | - | 0.86 (2039) | - | 0.82 (1154) |
| Coronary artery disease | - | 1 | - | 1 | - | 1 | - | 1 |
| eGFR (CKDEPI) | 80.2 (1498) | - | 79.6 (666) | - | 65.0 (1746) | - | 63.1 (1067) | - |
| hs-CRP |  | - |  | - |  | - |  | - |
| Current smoking | - | 0.15 (1237) | - | 0.12 (489) | - | 0.07 (1419) | - | 0.06 (757) |
| Cerebrovascular disease | - | 0 | - | 0 | - | 0 | - | 0 |
| Diabetes Mellitus | - | 0.25 (1686) | - | 0.21 (726) | - | 0.28 (2039) | - | 0.26 (1154) |
| Non HDL-cholesterol | 1.15 (1407) | - | 1.44 (726) | - | 1.21 (1539) | - | 1.5 (896) | - |
| Peripherial artery disease | - | 0.03 (1686) | - | 0.03 (726) | - | 0.06 (2039) | - | 0.05 (1154) |
| Systolic blood pressure | 125.4 (1686) | - | 124.6 (726) | - | 125.9 (2039) | - | 126.4 (1154) | - |
| Years since first ASCVD diagnosis | 9.0 (1686) | - | 7.7 (726) | - | 12.8 (2039) | - | 10.2 (1154) | - |

**Table S6. Mean risk factor values used as predictors in SMART2 model, for the subgroup with controlled hypertension entering the model with a history of Stroke**

|  | **Age 40-69**  **Males (n= 808)** | | **Age 40-69**  **Females (n= 858)** | | **Age 70+**  **Males (n= 1355)** | | **Age 70+**  **Females (n= 1275)** | |
| --- | --- | --- | --- | --- | --- | --- | --- | --- |
| **Predictor** | **Mean** | **Prop.** | **Mean** | **Prop.** | **Mean** | **Prop.** | **Mean** | **Prop.** |
| Abdominal aortic aneurysm | - | 0 | - | 0 | - | 0 | - | 0 |
| Age, years | 61.4 (808) | - | 59.9 (858) | - | 79.3 (1355) | - | 80.7 (1275) | - |
| Aspirin or equivalent | - | 0.85 (808) | - | 0.83 (858) | - | 0.88 (1355) | - | 0.85 (1275) |
| Coronary artery disease | - | 0.1 (808) | - | 0.05 (858) | - | 0.18 (1355) | - | 0.12 (1275) |
| eGFR (CKDEPI) | 79.6 (687) | - | 78.8 (784) | - | 64.7 (1166) | - | 64.8 (1275) | - |
| hs-CRP |  | - |  | - |  | - |  | - |
| Current smoking | - | 0.14 (573) | - | 0.17 (653) | - | 0.06 (964) | - | 0.06 (857) |
| Cerebrovascular disease | - | 1 | - | 1 | - | 1 | - | 1 |
| Diabetes Mellitus | - | 0.16 (808) | - | 0.14 (858) | - | 0.24 (1355) | - | 0.20 (1275) |
| Non HDL-cholesterol | 1.22 (655) | - | 1.5 (741) | - | 1.25 (1017) | - | 1.55 (984) | - |
| Peripherial artery disease | - | 0.02 (808) | - | 0.02 (858) | - | 0.06 (1355) | - | 0.03 (1275) |
| Systolic blood pressure | 125.94 (808) | - | 124.1 (858) | - | 126.4 (1355) | - | 126.3 (1275) | - |
| Years since first ASCVD diagnosis | 7.1 (808) | - | 8.0 (858) | - | 9.7 (1355) | - | 8.7 (1275) | - |

**Table S7. Mean risk factor values used as predictors in SMART2 model, for the subgroup with uncontrolled hypertension entering the model with a history of Stroke**

|  | **Age 40-69**  **Males (n= 577)** | | **Age 40-69**  **Females (n= 479)** | | **Age 70+**  **Males (n= 1294)** | | **Age 70+**  **Females (n= 1625)** | |
| --- | --- | --- | --- | --- | --- | --- | --- | --- |
| **Predictor** | **Mean** | **Prop.** | **Mean** | **Prop.** | **Mean** | **Prop.** | **Mean** | **Prop.** |
| Abdominal aortic aneurysm | - | 0 | - | 0 | - | 0 | - | 0 |
| Age, years | 61.5 (577) | - | 61.2 (479) | - | 79.7 (1294) | - | 80.0 (1625) | - |
| Aspirin or equivalent | - | 0.83 (577) | - | 0.80 (479) | - | 0.86 (1294) | - | 0.84 (1625) |
| Coronary artery disease | - | 0.11 (577) | - | 0.08 (479) | - | 0.19 (1294) | - | 0.11 (1625) |
| eGFR (CKDEPI) | 79.3 (577) | - | 78.5 (419) | - | 63.9 (1113) | - | 64.5 (1625) | - |
| hs-CRP |  | - |  | - |  | - |  | - |
| Current smoking | - | 0.16 (373) | - | 0.14 (301) | - | 0.06 (1294) | - | 0.05 (1002) |
| Cerebrovascular disease | - | 1 | - | 1 | - | 1 | - | 1 |
| Diabetes Mellitus | - | 0.19 (577) | - | 0.20 (479) | - | 0.24 (1294) | - | 0.20 (1625) |
| Non HDL-cholesterol | 1.20 (452) | - | 1.44 (479) | - | 1.27 (950) | - | 1.53 (1212) | - |
| Peripherial artery disease | - | 0.04 (577) | - | 0.02 (479) | - | 0.06 (1294) | - | 0.05 (1625) |
| Systolic blood pressure | 146.6 (577) | - | 147.5 (479) | - | 149.9 (1294) | - | 152.2 (1625) | - |
| Years since first ASCVD diagnosis | 7.2 (577) | - | 8.5 (479) | - | 10.0 (1294) | - | 8.8 (1625) | - |

**Table S8. Completed TECH-VER checklist of white-and black-box tests**

| **Test description (Please document how the test is conducted, as well)** | **Result of the test** |
| --- | --- |
| *Pre-analysis calculations* | |
| Does the technology (drug/device, etc.) acquisition costs increase with higher prices? | Yes |
| Does the drug acquisition cost increase for higher weight or body surface area? | NR |
| Does the probability of an event, derived from an odds ratio (OR)/ relative risk (RR) / hazard ratio (HR) and baseline probability, increases with higher OR/RR/HR? | No HR/OR are used, but in our case the probability is indeed affected by the effect of the intervention on BP. |
| If survival parametric distributions are used in the extrapolations, can the formulae used for the Weibull (generalized gamma) distribution generate the values obtained from the exponential (the Weibull or Gamma) distribution(s) under some parameter transformations? | NR |
| In a partitioned survival model, does the progression free survival curve or the time on treatment curve crosses the overall survival curve? | NR |
| If survival parametric distributions are used in the extrapolations or time-to-event calculations, can the formulae used for the Weibull (generalized gamma) distribution generate the values obtained from the exponential (the Weibull or Gamma) distribution(s) after replacing/transforming some of the parameters? | NR |
| Is hazard ratio calculated from Cox proportional hazards model applied on top of the parametric distribution extrapolation found from the survival regression? | NR |
| For the treatment effect inputs, if the model uses outputs from WINBUGs, are the OR, HR and RR values all within plausible ranges? (should be all non-negative and the average of these WINBUGs outputs should give the mean treatment effect) | NR |
| *Event-state calculations* | |
| Calculate the sum of the number of patients at each health state | Always 1 |
| Check if all probabilities and number of patients in a state are greater than or equal to zero | Yes |
| Check if all probabilities are smaller than or equal to one | Yes |
| Compare the number of dead (or any absorbing state) patients in a period with the number of dead (or any absorbing state) patients in the previous periods? | Compared, number always increases |
| In case of lifetime horizon, check if all patients are dead at the end of the time horizon | Yes |
| *Discrete event simulation specific:* sample one of the “time to event” types used in the simulation from the specified distribution. Plot the samples and compare the mean and the variance from the sample | NR |
| Set all utilities to one  Set all utilities to zero | When 1, total QALYs = number of cycles for both strategies (discounting at 0%).  When 0, total QALYs=0 for both strategies |
| Decrease all state utilities simultaneously (but keep event based utility decrements constant) | Lower utilities are accumulated each time |
| Set all costs to zero | Total costs = 0 for both strategies |
| Put mortality rates to 0 | Patients never die. |
| Put mortality rate extremely high | All patients die within first few cycles. |
| Set the effectiveness, utility and safety related model inputs for all treatment options equal | Mean life years and QALYs equal for both strategies. |
| In addition to the inputs above, set cost related model inputs for all treatment options equal | Mean costs, life years and QALYs equal for both strategies. |
| Change around the effectiveness, utility and safety related model inputs between two treatment options | Life years and QALYs reversed. |
| Check if the number of alive patients estimate at any cycle is in line with general population life table statistics | As we have excess deaths due to CVD and we adjust genpop mortality for CVD mortality, our mortality is always lower. |
| Check if the QALY estimate at any cycle is in line with general population utility estimates | Our utilities are always lower. |
| Set the inflation rate of the previous year higher | NR |
| Calculate the sum of all ingoing and outgoing transition probabilities | Sums to 1 |
| Calculate the number of patients entering and leaving a tunnel state throughout the time horizon | NR no tunnel states |
| Check if the time conversions for probabilities were conducted correctly. | Yes |
| *Decision tree specific:* calculate the sum of the expected probabilities of the terminal nodes | NR |
| *Patient-level model specific:* check if common random numbers are maintained for sampling for the treatment arms? | NR |
| *Patient-level model specific:* check if correlation in patient characteristics is taken into account when determining starting population? | NR |
| Increase the treatment acquisition cost | Higher total costs |
| *Population model specific:* set the mortality and incidence rates to zero | NR |
| *Result calculations* | |
| Check the incremental life years and QALYs gained results. Are they in line with the comparative clinical effectiveness evidence of the treatments involved? | Yes, treatment increases QALYs and LE due to a prevention of events. |
| Check the incremental cost results. Are they in line with the treatment costs? | Treatment is more costly |
| Total life years > total quality adjusted life years | Yes |
| Undiscounted results > discounted results | Yes |
| Divide undiscounted total QALYs by undiscounted life years. | Number is < max utility and > than min utility. |
| Subgroup analysis results: How do the outcomes change if the characteristics of the baseline change? | Total costs, QALYs, and LEs most favorable for healthiest subgroup and least favorable for sickest subgroup. |
| Could you generate all the results in the report from the model (including the uncertainty analysis results)? | Nearly all, but results about the efficiency gains in time spent on care relied on separate calculations as well. |
| Does the total life years, QALYs and costs decrease if a shorter time horizon is selected? | Yes |
| Is the reporting and contextualization of the incremental results correct? | Only ‘cost-effective’ is applicable in this study. |
| Are the reported ICERs in the fully incremental analysis non-decreasing? | NR, only SOC and proposed strategy |
| If disentangled results are presented, do they sum up to the total results? (e.g. different cost types sum up to the total costs estimate) | Yes |
| Check if half cycle correction is implemented correctly (total life years with half cycle correction should be lower than without) | Yes |
| Check the discounted value of costs/QALYs after 2 years | Discounted value=undiscounted/(1+r)2 |
| Set discount rates to zero | Discounted and undiscounted values equal |
| Set mortality rate to zero | Total LE = number of cycles |
| Put the consequence of adverse event/discontinuation to zero. (zero costs and zero mortality/utility decrements) | When effect of intervention = 0 and Pr of CVD death (from any state) = 0, then:  The results when consequences of MI/stroke = 0 are the same as the results when Pr of MI/stroke = 0. |
| Divide total undiscounted treatment acquisition costs by the average duration on treatment. | Similar to annual costs under strategy. |
| Set discount rates to a higher value | Lower costs and QALYs |
| Set discount rates of costs/effects to an extremely high value | Total discounted results are similar to the discounted results accrued in the first cycles |
| Put adverse event/discontinuation rates to zero and then to extremely high level. | Less costs higher QALYS/LYs when MI/stroke rates are 0, higher costs and lower QALYS/LYs when these rates are 5xhigher. |
| Double the difference in efficacy and safety between new intervention and comparator and report the incremental results. | Incremental QALYs base case = 0.05, with double difference in effectiveness QALYs = 0.1. |
| Do the same for a scenario in which the difference in efficacy and safety is halved. | Incremental QALYs base case = 0.05, with half difference in effectiveness QALYs = 0.03. |
| *Uncertainty analysis calculations* | |
| Are all parameters subject to uncertainty included in the one-way sensitivity analysis (OWSA)?  Check if the OWSA includes any parameters associated with joint uncertainty (e.g. parts of a utility regression equation, survival curves with multiple parameters). | Yes  No |
| Are the upper and lower bounds used in the one-way sensitivity analysis used confidence intervals based on the statistical distribution assumed for that parameter?  Are the resulting ICER, incremental costs/QALYs with upper and lower bound of a parameter plausible and in line with a priori expectations? | No, upper and lower bounds equal to +/- 25% of base value, unless such a bound is not reasonable (e.g., utilities >1) or in line with a priori expectations. |
| Check that all parameters used in the sensitivity analysis have an appropriate associated distributions  - upper and lower bounds should surround the deterministic value (i.e. Upper bound ≥ mean ≥ Lower bound)  - standard error and not standard deviation used in sampling  - Lognormal / gamma distribution for hazard ratios and costs/ resource use  - Beta for utilities and proportions/probabilities  - Dirichlet for multinomial  - Multivariate normal for correlated inputs (e.g. survival curve or regression parameters)  - Normal for other variables as long as samples don’t violate requirement to remain positive when appropriate | In general yes, although we opted to use triangular distributions for many parameters that were informed by various sources of literature. |
| Check PSA output mean costs, QALYs and ICER compared to the deterministic results. Is there a large discrepancy? | NR, main analysis was probabilistic. |
| If you take new PSA runs from the excel model do you get similar results? | NR, we used an R model and get exactly the same results. |
| Is(are) the CEAC line(s) in line with the CE scatter plots and the efficient frontier? | Yes |
| Does the PSA cloud demonstrate an unexpected behavior or has an unusual shape? | No |
| Is the sum of all CEAC lines equal to 1 for all WTP values? | Yes |
| Are the explored scenario analyses provide a balanced view on the structural uncertainty? (i.e. not always looking at more optimistic scenarios) | Yes |
| Are the scenario analysis results plausible and in line with a priori expectations? | Yes |
| Check the correlation between 2 PSA results (i.e. costs/QALYs under the SoC and costs/QALYs under the comparator) | High correlation because most parameters use the same random streams for different arms |
| If a certain seed is used for random number generation (or previously generated random numbers are used), check if they are they scattered evenly between 0-1 when they are plotted? | NR |
| Compare the mean of the parameter samples generated by the model against the point estimate for that parameter, use graphical methods to examine distributions, functions | The sample means and the point estimates are similar and the graphs are consistent with the corresponding distribution functions (e.g. Normal, Gamma, etc.) |
| Check if sensitivity analyses include any parameters associated with methodological/ structural uncertainty (e.g. annual discount rates, time horizon). | No |
| Value of information analysis if applicable: Was this implemented correctly?  Which types of analysis? Were aggregated parameters used? Which parameters are grouped together? Does it match the write-up’s suggestions?  Is EVPI larger than all individual EVPPI?  Is EVPPI for a (group of) parameters larger than the EVSI of that (group) of parameter(s)?  Are the results from EVPPI in line with OWSA or other parameter importance analysis (e.g. ANCOVA)? | NR |
| Did the electronic model pass the black-box tests of the previous verification stages in all PSA iterations and in all scenario analysis settings? (additional macro can be embedded to PSA code, which stops the PSA when an error such as negative transition probability, is detected) | Yes |
| OWSA=one-way sensitivity analysis; ICER = incremental cost-effectiveness ratio; PSA = probabilistic sensitivity analysis; WTP = willingness to pay; CE = cost-effectiveness; CEAC = cost-effectiveness acceptability curve; LY = life years; QALYs = Quality adjusted life years; OR = odds ratio; RR= relative risk; HR = hazard ratio | |

**Table S9. Scenario analysis results. Costs, QALYs, and incremental cost-effectiveness ratios (ICERs) vs care as usual.**

| Scenario | Costs  Mean (95% CI) | | | QALYs  Mean (95% CI) | | | ICER |
| --- | --- | --- | --- | --- | --- | --- | --- |
|  | CaU | Intervention | CaU | | Intervention |  | |
| Healthcare perspective | | | | | | | |
| *Controlled, no history* | 6218 (4256, 8431) | 7131 (4960, 9439) | 20.66 (19.66, 21.61) | | 20.68 (19.68, 21.63) | 42971 | |
| *Controlled, history of MI/stroke* | 90538 (63338, 120369) | 91548 (64820, 121417) | 12.28 (10.34, 13.98) | | 12.38 (10.41, 14.12) | 10523 | |
| *Uncontrolled, no history* | 7879 (5369, 10730) | 8853 (6218, 11704) | 18.74 (17.38, 19.98) | | 18.79 (17.42, 20.01) | 16330 | |
| *Uncontrolled, history of MI/stroke* | 92542 (64159, 123850) | 94192 (66664, 125263) | 9.86 (7.56, 11.89) | | 10.07 (7.74, 12.15) | 7651 | |
| *Pooled* | 21476 (14964, 28656) | 22459 (16048, 29691) | 18.43 (17.3, 19.52) | | 18.48 (17.34, 19.58) | 18434 | |
| No discounting | | | | | | | |
| *Controlled, no history* | 12990 (8904, 17518) | 14641 (10298, 19378) | 26.51 (25.23, 27.73) | | 26.54 (25.26, 27.77) | 49848 | |
| *Controlled, history of MI/stroke* | 151322 (108498, 198743) | 153627 (111492, 201180) | 14.7 (12.35, 16.77) | | 14.83 (12.44, 16.98) | 17569 | |
| *Uncontrolled, no history* | 15925 (10901, 21502) | 18236 (12791, 23942) | 23.94 (22.2, 25.53) | | 24.03 (22.26, 25.58) | 25259 | |
| *Uncontrolled, history of MI/stroke* | 151005 (108139, 198678) | 155340 (113157, 203141) | 11.6 (8.86, 14.02) | | 11.89 (9.1, 14.38) | 15174 | |
| *Pooled* | 37918 (26875, 50171) | 40010 (28931, 52411) | 23.43 (21.99, 24.81) | | 23.5 (22.06, 24.89) | 26882 | |
| Starting age of 40 years | | | | | | | |
| *Controlled, no history* | 6836 (4979, 8921) | 7781 (5661, 10048) | 25.14 (23.92, 26.29) | | 25.16 (23.95, 26.32) | 42100 | |
| *Controlled, history of MI/stroke* | 117678 (85466, 153112) | 118488 (86715, 153816) | 15.47 (13, 17.63) | | 15.59 (13.09, 17.81) | 6991 | |
| *Uncontrolled, no history* | 8992 (6524, 11800) | 9857 (7201, 12699) | 22.86 (21.21, 24.37) | | 22.92 (21.25, 24.4) | 13337 | |
| *Uncontrolled, history of MI/stroke* | 123041 (89155, 160530) | 124308 (91126, 161772) | 12.48 (9.54, 15.07) | | 12.75 (9.76, 15.41) | 4707 | |
| *Pooled* | 27018 (19718, 35112) | 27935 (20621, 36094) | 22.53 (21.14, 23.87) | | 22.59 (21.19, 23.94) | 15087 | |
| Intervention effect reduces by 40% after 5 years | | | | | | | |
| *Controlled, no history* | 6821 (4820, 9076) | 7672 (5482, 10023) | 20.66 (19.66, 21.61) | | 20.67 (19.68, 21.63) | 63664 | |
| *Controlled, history of MI/stroke* | 99572 (71692, 130026) | 100253 (72687, 130647) | 12.28 (10.34, 13.98) | | 12.35 (10.39, 14.1) | 10453 | |
| *Uncontrolled, no history* | 8706 (6144, 11615) | 9497 (6826, 12404) | 18.74 (17.38, 19.98) | | 18.77 (17.41, 20) | 20921 | |
| *Uncontrolled, history of MI/stroke* | 103878 (74641, 135967) | 104655 (75888, 136662) | 9.86 (7.56, 11.89) | | 10.01 (7.68, 12.08) | 5214 | |
| *Pooled* | 23728 (17038, 31066) | 24534 (17847, 31914) | 18.43 (17.3, 19.52) | | 18.46 (17.32, 19.57) | 23110 | |

**Table S10. Annual expected reductions in workload with the intervention, per GP and PN and by subgroups of controlled and uncontrolled BP.**

|  | **Controlled BP** | | | **Uncontrolled BP** | | |
| --- | --- | --- | --- | --- | --- | --- |
|  | **Intervention** | **CaU** | **Increase (+) or reduction (-) with CVRM-box** | **Intervention** | **CaU** | **Increase (+) or reduction (-) with CVRM-box** |
| **Long patient contacts**^a^ |  |  |  |  |  |  |
| Hours spent per GP, annually | 2.80 | 2.33 | 0.47 | 1.82 | 1.82 | 0.00 |
| Hours spent per PN, annually | 91.92 | 86.86 | 5.06 | 65.79 | 60.31 | 5.48 |
| **Short patient contacts**^b^ |  |  |  |  |  |  |
| Hours spent per GP, annually | 1.63 | 1.87 | -0.23 | 1.21 | 1.82 | -0.61 |
| Hours spent per PN, annually | 52.71 | 67.46 | -14.76 | 41.94 | 63.6 | -21.66 |
| **Remote consultations** |  |  |  |  |  |  |
| Frequency, per GP, annually | 4.20 | 5.60 | -1.40 | 2.73 | 4.55 | -1.82 |
| Frequency, per PN, annually | 101.19 | 113.00 | -11.81 | 65.79 | 98.69 | -32.90 |
| ^a^ duration of 20 minutes with GP and 30 minutes with PN  ^b^ duration of 10 minutes with GP and 15 minutes with PN  Note: ‘hours’ and ‘consultations’ refer to *annual* amounts per GP or PN in a reference primary care practice assuming an eligible cardiovascular population of N=231 patients per practice (supplementary Methods) | | | | | | |

**Figure S1. Budget impact analysis results assuming scale-up at regional and national levels**

(A) Intervention budget impact, calculated as the difference in total costs between intervention (C) and CaU (B) over the 5-year time horizon of the budget impact analysis. Note that negative values mean cost savings.
