# Supplementary material for: A Cardiovascular Care Pathway Supported by Remote Patient Management in Dutch Primary Care: Cost-Effectiveness, Budget Impact, and Workload Analysis
Source: JMIR Mhealth Uhealth. 2026 Sep 28;14:e79512. doi: 10.2196/79512 (PMC13618991; doi:10.2196/79512)
Supplement: Supplementary file 2 [file mhealth-v14-e79512-s002.docx]

# Appendix 2: Methodological details for main analysis, budget impact analysis, and workload reduction calculations

**Transition probabilities and event occurrence**

The SCORE2, SCORE2-OP, and SMART2 prediction equations were populated with subgroup-specific mean risk factor values (e.g., cholesterol, smoking status, BP etc., reported in Tables 2-7) estimated from the ELAN population, which is representative of care as usual. As the SCORE2 model was intended for the prediction of a 10-year risk, the age predictor was updated every ten cycles. Conversely, SMART2 enables the calculation of a one-year risk; thus, the age predictor was updated in every cycle. All other risk factor values were updated when the cohort reached age 70 to account for age differences in risk factors and for consistency with the recommended use of SCORE2-OP.

As the SCORE2 models predict cumulative risks of a composite (i.e., MACE) endpoint over a time period of 10 years ($R_{10}$), these risks were converted to annual rates as $r= -\left( \frac{1}{10} \right)\times\ln\left( 1-R_{10} \right)$and then to annual probabilities as $p= 1-e^{\left( -r1 \right)}.$ These resulting composite probabilities were then disaggregated into event-specific probabilities using publicly available frequencies/distributions of non-fatal MI, non-fatal stroke, and fatal CVD events in the Netherlands by age groups (shown in the table below). The same procedure was followed with the SMART2 predicted 1-year risk of MACE.

**Distributions of non-fatal MI, non-fatal stroke, and fatal CVD events in the Netherlands by age groups.**

|  | Ages  20-45yrs | Ages  45-65yrs | Ages  65-80yrs | Age  80y+ |
| --- | --- | --- | --- | --- |
| Number of non-fatal MI men | 875 | 9990 | 10330 | 4025 |
| Number of non-fatal stroke men | 560 | 5355 | 10120 | 5945 |
| Number of fatal event men | 172 | 1884 | 5732 | 8915 |
| Number of non-fatal MI women | 325 | 3230 | 4890 | 3545 |
| Number of non-fatal stroke women | 545 | 3175 | 7235 | 7760 |
| Number of fatal event women | 63 | 777 | 3436 | 12307 |

The above table shows the total number of events in the Netherlands in 2021. For non-fatal events, the total number of hospitalizations were used (from: <https://opendata.cbs.nl/statline/#/CBS/nl/dataset/84069NED/table>), applying the same ICD-10 classifications as the SCORE2 and SMART2 models. The number of fatal events were obtained from <https://opendata.cbs.nl/statline/#/CBS/nl/dataset/7233/table?ts=1722329174524>, also applying the ICD-10 classifications as included in SCORE2 and SMART2. These numbers were used to calculate the probability of a MACE event (as predicted by SCORE2 and SMART2) being a non-fatal MI, non-fatal stroke, or fatal CVD event.

**Corrections of general population mortality for CVD-related mortality**

Annual probabilities of all-cause mortality were estimated from general population lifetables and correcting for mortality due to fatal CVD events (which is accounted for separately as described above). Specifically, the proportion of CVD deaths out of all deaths in the Netherlands on 2021 was extracted from publicly available data [28]. The ICD-10 codes used to count the number of CVD deaths were consistent with those used for SCORE2 and SMART2 (i.e., I10, I11, I12, I13, I15, I20, I21, I22, I23, I24, I25, I46, I47, I48, I49, I50, I51, I52, I61, I63, I64, I65, I66, I67, I68, I69, I70, I71, I72, I73, R96). The resulting proportions were used as a multiplication factor to correct the annual probabilities of all-cause death (from lifetables) to exclude CVD deaths.

**Costs**

Our modelling approach integrates state- and transition-specific costs (Table 1 and supplementary Table 1). State-specific costs represent the annual costs associated with each health state, excluding any one-time short-term costs that would arise from transitioning to that state (i.e., costs associated with the acute stroke/MI events themselves). To address the latter, transition-specific costs representing the high, short-term costs associated with MACE were included. Transition costs were counted only once per transition by keeping track of the cohort's transition dynamics (i.e., the proportion of patients transitioning between any two states in each cycle, see [29]), while state costs were applied recurringly per cycle according to the proportion of patients in each state.

Annual intervention and CaU costs were estimated using data from the prospective study of CVRM-Box and ELAN data, respectively, differentiating between controlled and uncontrolled BP patients (Table 1). Additionally, patients in any post-MI or post-stroke states (including subgroups with a history of MI/stroke entering the model in those states) accrued an incremental annual cost sources from the academic literature (Table 1 and supplementary Table 1).

Non-healthcare costs included work productivity losses, which were accrued only during the cycles when the cohort was of usual working age in the Netherlands (i.e., until 67 years old). Several studies have shown that the period of work absence after an MI or stroke event can vary greatly, but is often around 3-4 months [5,9,13–17]. Therefore, in line with the friction method for calculating productivity losses, we applied productivity losses as transition costs instead of (annual) state costs. These transition costs were only accrued on the first transition to a worse health state to prevent double counting (i.e., friction costs were only accrued once).

**Assumptions**

As with every model, ours is subject to simplifying assumptions:

- There is no waning in effect nor change in adherence to the intervention. In practice, reductions in adherence or treatment discontinuation would be expected. To address this, we explore a scenario whereby patient’s BP and smoking rate increases again after 5 years. The waning effect (especially in the long-term) is difficult to estimate in practice, especially since a proportion of patients who stop adhering may do so due to positive effects of the intervention and self-management. In this study, we assumed that the effectiveness of treatment on BP levels and smoking rates reduces by 40% after 5 years, based on data from the CVRM-Box prospective study.
- Patients can only experience one recurrent event of the same type (Figure 1). Although third/fourth events may occur in practice, these are much less frequent [65,66] and evidence to inform corresponding parameters is lacking. Even with assumed parameters, the costs and dis-utilities associated with a low number of 3^rd^ and 4^th^ events (which additionally occur in later model cycles and are therefore heavily discounted), is unlikely to influence results.
- The costs of a fatal CVD event are equivalent to the lowest of the non-fatal events, to avoid overestimating the benefit of preventing CVD deaths. This parameter had to be assumed as we identified no suitable studies to inform this parameter alternatively.

**Budget impact analyses**

To produce the reported 5-year budget impact results, the following steps were taken.

1. The model was ran for 5 cycles with no discounting and including only healthcare costs. The costs for each cycle were saved.
2. The costs for each cycle were multiplied by the eligible population sizes (see table below) for the number of cycles during which they were assumed to receive the intervention (or usual care). For example, at the regional level, the 8344 patients entering in year 1 are assumed to receive the intervention for 5 years, so they accrue the intervention costs for cycles 1 to 5. In contrast, the 528 patients entering in year 3 only accrue the costs for cycles 1 to 3. The total accrued costs with intervention (or usual care) are the sum of: all the cycle costs multiplied by the applicable population sizes throughout the 5-year period.
3. The intervention budget impact was calculated as the difference between a) the total accrued costs assuming the eligible population receives the intervention and b) the total accrued costs assuming the same eligible population receives usual care.

**Eligible population sizes per year during the first 5-year rollout period.**

|  | Regional level | | National level | |
| --- | --- | --- | --- | --- |
| Year | Eligible N | New eligible N | Eligible N | New eligible N |
| 1 | 8344 | 0 | 1917812 | 0 |
| 2 | NA | 501 | NA | 113493 |
| 3 | NA | 528 | NA | 125002 |
| 4 | NA | 392 | NA | 90140 |
| 5 | NA | 492 | NA | 114173 |

Assumptions in budget impact analysis

1. The characteristics of patients entering each year are identical to the patients modelled in the main analysis (e.g., in terms of relative subgroup sizes, MACE risk factor values used for SCORE2 and SMART2 risk prediction, age, and proportion of males).

Notes

1. The eligible population sizes for the regional level rollout correspond to primary data from InEen [30] on the total number of patients in the integrated care path for cardiovascular diseases in the region of South-Holland-North. From those totals, we assumed that 55% were eligible to receive the intervention (or usual care).
2. The eligible population sizes for the national level rollout correspond to publicly available data from NIVEL [31] on the total number of people in the same integrated care path for cardiovascular diseases as in the South-Holland-North region. From those totals, we assumed that 55% were eligible to receive the intervention (or usual care).

**Workload reduction calculations**

The below calculation was be repeated for short and long visits with GPs and PNs, by subgroups of controlled BP and uncontrolled BP (i.e., in total 6 times).

$$H_{r}=\frac{D_{r}\left( F_{rs}-F_{ri} \right)N_{p}}{N_{prac}} eq. 1$$

Where

$H_{r}$= annual number of hours saved on resource $r$ per practitioner (i.e., GP or PN)

$r$ = resource category (i.e., short or long visits with GP or PN)

$D_{r}$ = assumed duration of visit, in hours, for resource group $r$

$F_{ri}$ = mean frequency of resource use $r$per year, among patients receiving the intervention as observed in the prospective study of CVRM-Box.

$F_{rs}$ = mean frequency of resource use $r$per year, among (matched) patients receiving standard care, as observed in the prospective study of CVRM-Box.

$N_{p}$ = patient population size multiplier for a reference primary care practice (see assumptions below).

$N_{prac}$ = Number of practitioners (GPs or PNs, depending on the calculation) per reference primary care practice.

For the reduction in total number of e-consultations per year, the calculation simplifies to:

$$E_{prac}=\left( F_{es}-F_{ei} \right)N_{p} eq. 2$$

Where

$E_{prac}$= annual number of e-consultations saved for provider $prac$

$prac$ = practitioner (either PN or GP)

$F_{ei}$ = mean number of e-consultations per patient per year with intervention as observed in the prospective study of CVRM-Box.

$F_{es}$ = mean number of e-consultations per patient per year with standard care as observed in the prospective study of CVRM-Box.

$N_{p}$ = patient population size multiplier for a reference primary care practice (see Notes below).

Data source

Primary data from the prospective matched cohort study were used to estimate $F_{ri}$ and $F_{rs}$ (eq 1) and $F_{i}$ and $F_{s}$ (eq. 2). Specifically, the following data was available.

|  | Intervention | | Usual care | |
| --- | --- | --- | --- | --- |
|  | Controlled BP | Uncontrolled BP | Controlled BP | Uncontrolled BP |
| Mean number of long contacts with GP or PN per patient per year | 1.15 | 1.26 | 1.08 | 1.16 |
| Mean number of short contacts with GP or PN per patient per year | 1.32 | 1.61 | 1.68 | 2.44 |
| Mean number of remote consultations with GP or PN per patient per year | 0.63 | 0.63 | 0.71 | 0.95 |

Notes

1. Short GP visits are scheduled to last 10 minutes, in line with actual scheduling practices.
2. Long GP visits are scheduled to last 20 min, in line with actual scheduling practices.
3. Short PN visits are scheduled to last 15 min, in line with actual scheduling practices.
4. Long PN visits are scheduled to last 30 min, in line with actual scheduling practices.
5. Mean number of patients per reference practice = 2100 [30,32]
   a. Of which constitute a CVD population = 420 [30]
   b. Of which are assumed eligible to receive the intervention or CaU: 55%
6. Number of GPs per primary care practice = 1 [32]
7. Number of PNs per primary care practice = 0.83 [32]

Assumptions

1. The ratio of GP to PN visits and/or e-consultations (from the above table): 5:95.

The proportions of controlled and uncontrolled BP patients are consistent with the main analysis (i.e., 57% and 43% respectively).

**eReferences**

[1]	McManus RJ, Mant J, Franssen M, Nickless A, Schwartz C, Hodgkinson J, et al. Efficacy of self-monitored blood pressure, with or without telemonitoring, for titration of antihypertensive medication (TASMINH4): an unmasked randomised controlled trial. The Lancet 2018;391:949–59. https://doi.org/10.1016/S0140-6736(18)30309-X.

[2]	Greving JP, Visseren FLJ, De Wit GA, Algra A. Statin treatment for primary prevention of vascular disease: whom to treat? Cost-effectiveness analysis. Bmj 2011;342.

[3]	Janzon M, Henriksson M, Hasvold P, Hjelm H, Thuresson M, Jernberg T. Long-term resource use patterns and healthcare costs after myocardial infarction in a clinical practice setting: results from a contemporary nationwide registry study. European Heart Journal–Quality of Care and Clinical Outcomes 2016;2:291–8.

[4]	Kolominsky-Rabas PL, Heuschmann PU, Marschall D, Emmert M, Baltzer N, Neundörfer B, et al. Lifetime cost of ischemic stroke in Germany: results and national projections from a population-based stroke registry: the Erlangen Stroke Project. Stroke 2006;37:1179–83.

[5]	van Mastrigt G, van Heugten C, Visser-Meily A, Bremmers L, Evers S. Estimating the Burden of Stroke: Two-Year Societal Costs and Generic Health-Related Quality of Life of the Restore4Stroke Cohort. Int J Environ Res Public Health 2022;19. https://doi.org/10.3390/ijerph191711110.

[6]	Cowper PA, Knight JD, Davidson‐Ray L, Peterson ED, Wang TY, Mark DB, et al. Acute and 1‐year hospitalization costs for acute myocardial infarction treated with percutaneous coronary intervention: results from the TRANSLATE‐ACS registry. J Am Heart Assoc 2019;8:e011322.

[7]	Soekhlal RR, Burgers LT, Redekop WK, Tan SS. Treatment costs of acute myocardial infarction in the Netherlands. Netherlands Heart Journal 2013;21:230–5.

[8]	Sülz S, Wagenaar H, Van de Klundert J. Have Dutch Hospitals Saved Lives and Reduced Costs? A longitudinal patient‐level analysis over the years 2013–2017. Health Econ 2021;30:2399–408.

[9]	Van Eeden M, Van Heugten C, Van Mastrigt G, Van Mierlo M, Visser-Meily JMA, Evers S. The burden of stroke in the Netherlands: estimating quality of life and costs for 1 year poststroke. BMJ Open 2015;5:e008220.

[10]	Geessinck FAJ, Pleijhuis RG, Mentink RJ, van der Palen J, Koffijberg H. Cost-effectiveness analysis of the DiagnOSAS screening tool compared with polysomnography diagnosis in Dutch primary care. Journal of Clinical Sleep Medicine 2018;14:1005–15.

[11]	Buisman LR, Tan SS, Nederkoorn PJ, Koudstaal PJ, Redekop WK. Hospital costs of ischemic stroke and TIA in the Netherlands. Neurology 2015;84:2208–15.

[12]	Baeten SA, van Exel NJA, Dirks M, Koopmanschap MA, Dippel DWJ, Niessen LW. Lifetime health effects and medical costs of integrated stroke services-a non-randomized controlled cluster-trial based life table approach. Cost Effectiveness and Resource Allocation 2010;8:1–10.

[13]	Steen Carlsson K, Nilsson K, Wolden ML, Faurby M. Economic burden of atherosclerotic cardiovascular disease: a matched case–control study in more than 450,000 Swedish individuals. BMC Cardiovasc Disord 2023;23:483.

[14]	Gordois AL, Toth PP, Quek RGW, Proudfoot EM, Paoli CJ, Gandra SR. Productivity losses associated with cardiovascular disease: a systematic review. Expert Rev Pharmacoecon Outcomes Res 2016;16:759–69.

[15]	Isaaz K, Coudrot M, Sabry MH, Cerisier A, Lamaud M, Robin C, et al. Return to work after acute ST-segment elevation myocardial infarction in the modern era of reperfusion by direct percutaneous coronary intervention. Arch Cardiovasc Dis 2010;103:310–6.

[16]	Bhattacharyya MR, Perkins-Porras L, Whitehead DL, Steptoe A. Psychological and clinical predictors of return to work after acute coronary syndrome. Eur Heart J 2007;28:160–5.

[17]	Sørensen J, Ploug UJ. The cost of diabetes‐related complications: registry‐based analysis of days absent from work. Economics Research International 2013;2013:618039.

[18]	Versteegh M, M. Vermeulen K, M. A. A. Evers S, de Wit GA, Prenger R, A. Stolk E. Dutch Tariff for the Five-Level Version of EQ-5D. Value in Health 2016;19:343–52. https://doi.org/10.1016/j.jval.2016.01.003.

[19]	Nyman JA, Barleen NA, Dowd BE, Russell DW, Coons SJ, Sullivan PW. Quality-of-life weights for the US population: self-reported health status and priority health conditions, by demographic characteristics. Med Care 2007;45:618–28.

[20]	Sullivan PW, Lawrence WF, Ghushchyan V. A national catalog of preference-based scores for chronic conditions in the United States. Med Care 2005;43:736–49.

[21]	Sullivan PW, Slejko JF, Sculpher MJ, Ghushchyan V. Catalogue of EQ-5D scores for the United Kingdom. Medical Decision Making 2011;31:800–4. https://doi.org/10.1177/0272989X11401031.

[22]	Luengo-Fernandez R, Gray AM, Bull L, Welch S, Cuthbertson F, Rothwell PM, et al. Quality of life after TIA and stroke: ten-year results of the Oxford Vascular Study. Neurology 2013;81:1588–95.

[23]	Xie J, Wu EQ, Zheng Z-J, Croft JB, Greenlund KJ, Mensah GA, et al. Impact of stroke on health-related quality of life in the noninstitutionalized population in the United States. Stroke 2006;37:2567–72.

[24]	Rebchuk AD, O’Neill ZR, Szefer EK, Hill MD, Field TS. Health utility weighting of the modified Rankin scale: a systematic review and meta-analysis. JAMA Netw Open 2020;3:e203767–e203767.

[25]	Lewis EF, Li Y, Pfeffer MA, Solomon SD, Weinfurt KP, Velazquez EJ, et al. Impact of cardiovascular events on change in quality of life and utilities in patients after myocardial infarction: a VALIANT study (valsartan in acute myocardial infarction). JACC Heart Fail 2014;2:159–65.

[26]	Munyombwe T, Hall M, Dondo TB, Alabas OA, Gerard O, West RM, et al. Quality of life trajectories in survivors of acute myocardial infarction: a national longitudinal study. Heart 2020;106:33–9.

[27]	Matza LS, Stewart KD, Gandra SR, Delio PR, Fenster BE, Davies EW, et al. Acute and chronic impact of cardiovascular events on health state utilities. BMC Health Serv Res 2015;15:1–11.

[28]	Netherlands Bureau of Statistics (CBS). StatLine: Overledenen; doodsoorzaak (uitgebreide lijst), leeftijd, geslacht 2021. https://opendata.cbs.nl/statline/#/CBS/nl/dataset/7233/table?ts=1726237214657 (accessed February 13, 2024).

[29]	Krijkamp EM, Alarid-Escudero F, Enns EA, Pechlivanoglou P, Hunink MGM, Yang A, et al. A multidimensional array representation of state-transition model dynamics. Medical Decision Making 2020;40:242–8.

[30]	InEen. InEen Benchmark Ketenzorg 2023 2023. https://ineen.nl/wp-content/uploads/2024/07/InEen-Benchmark-Ketenzorg-2023.pdf (accessed September 16, 2024).

[31]	Nivel Zorgregistraties eerste lijn. Hart- en vaatziekten | Leeftijd en geslacht 2023. https://www.vzinfo.nl/onderwerpen/hart-en-vaatziekten/leeftijd-en-geslacht (accessed September 16, 2024).

[32]	Heiligers PJM. Kennisvraag: praktijkondersteuners in de huisartspraktijk (POH’s) klaar voor de toekomst? Nivel; 2012.
