# Supplementary material for: A Cardiovascular Care Pathway Supported by Remote Patient Management in Dutch Primary Care: Cost-Effectiveness, Budget Impact, and Workload Analysis
Source: JMIR Mhealth Uhealth. 2026 Sep 28;14:e79512. doi: 10.2196/79512 (PMC13618991; doi:10.2196/79512)
Supplement: Supplementary file 3 [file mhealth-v14-e79512-s003.docx]

| **CHEERS CHECKLIST COMPLETED** | | | |
| --- | --- | --- | --- |
| **Section/topic** | **Item no.** | **Guidance for reporting** | **Reported in section** |
| Title | | |  |
| Title | 1 | Identify the study as an economic evaluation and specify the interventions being compared | Title page |
| Abstract | | |  |
| Abstract | 2 | Provide a structured summary that highlights context, key methods, results, and alternative analyses | Abstract |
| Introduction | | |  |
| Background and objectives | 3 | Give the context for the study, the study question, and its practical relevance for decision making in policy or practice | Introduction |
| Methods | | |  |
| Health economic analysis plan | 4 | Indicate whether a health economic analysis plan was developed and where available | not applicable |
| Study population | 5 | Describe characteristics of the study population (such as age range, demographics, socioeconomic, or clinical characteristics) | Methods subsection ‘Population and setting’ |
| Setting and location | 6 | Provide relevant contextual information that may influence findings | Methods subsection ‘Population and setting’ |
| Comparators | 7 | Describe the interventions or strategies being compared and why chosen | Methods subsection ‘Interventions’ |
| Perspective | 8 | State the perspective(s) adopted by the study and why chosen | Methods subsection ‘Costs’ |
| Time horizon | 9 | State the time horizon for the study and why appropriate | Methods subsection ‘Model structure’ |
| Discount rate | 10 | Report the discount rate(s) and reason chosen | Methods subsection ‘Costs’ and ‘Outcomes’ |
| Selection of outcomes | 11 | Describe what outcomes were used as the measure(s) of benefit(s) and harm(s) | Methods subsection ‘Outcomes’ |
| Measurement of outcomes | 12 | Describe how outcomes used to capture benefit(s) and harm(s) were measured | Methods subsection ‘Outcomes’ |
| Valuation of outcomes | 13 | Describe the population and methods used to measure and value outcomes | Methods subsection ‘Outcomes’ (Appendix 1) |
| Measurement and valuation of resources and costs | 14 | Describe how costs were valued | Methods subsection ‘Costs’ and Appendix 1. |
| Currency, price date, and conversion | 15 | Report the dates of the estimated resource quantities and unit costs, plus the currency and year of conversion | Table 1 and Appendix 1. |
| Rationale and description of model | 16 | If modelling is used, describe in detail and why used. Report if the model is publicly available and where it can be accessed | Methods subsection ‘Study design’ and ‘Model structure’. |
| Analytics and assumptions | 17 | Describe any methods for analysing or statistically transforming data, any extrapolation methods, and approaches for validating any model used | Methods subsections ‘Assumptions’ and ‘Model validation’ |
| Characterising heterogeneity | 18 | Describe any methods used for estimating how the results of the study vary for subgroups | All Methods |
| Characterising distributional effects | 19 | Describe how impacts are distributed across different individuals or adjustments made to reflect priority populations | not applicable given model-based analysis |
| Characterising uncertainty | 20 | Describe methods to characterise any sources of uncertainty in the analysis | Methods subsection ‘Uncertainty analyses’ |
| Approach to engagement with patients and others affected by the study | 21 | Describe any approaches to engage patients or service recipients, the general public, communities, or stakeholders (such as clinicians or payers) in the design of the study | not applicable given model-based design. |
| Results | | |  |
| Study parameters | 22 | Report all analytic inputs (such as values, ranges, references) including uncertainty or distributional assumptions | Table 1 and Supplementary table S1 |
| Summary of main results | 23 | Report the mean values for the main categories of costs and outcomes of interest and summarise them in the most appropriate overall measure | Table 2 |
| Effect of uncertainty | 24 | Describe how uncertainty about analytic judgements, inputs, or projections affect findings. Report the effect of choice of discount rate and time horizon, if applicable | Figure 2 and Table 2 |
| Effect of engagement with patients and others affected by the study | 25 | Report on any difference patient/service recipient, general public, community, or stakeholder involvement made to the approach or findings of the study | not applicable given model-based design. |
| Discussion | | |  |
| Study findings, limitations, generalisability, and current knowledge | 26 | Report key findings, limitations, ethical or equity considerations not captured and how these could affect patients, policy, or practice | Discussion section |
| Other relevant information | | | |
| Source of funding | 27 | Describe how the study was funded and any role of the funder in the identification, design, conduct, and reporting of the analysis | Funding statement (end of manuscript) |
| Conflicts of interest | 28 | Report authors’ conflicts of interest according to journal or International Committee of Medical Journal Editors requirements | Conflict of interest statement (end of manuscript) |
